# Dynamic changes of phenotype and function of natural killer cells in peripheral blood before and after thermal ablation of hepatitis B associated hepatocellular carcinoma and their correlation with tumor recurrence

**DOI:** 10.1186/s12885-023-10823-4

**Published:** 2023-05-30

**Authors:** Hai-Yan Wang, Xiong-Wei Cui, Yong-Hong Zhang, Yu Chen, Ning-Ning Lu, Li Bai, Zhong-Ping Duan

**Affiliations:** 1 Center of Interventional Oncology and Liver Diseases, Beijing Youan Hospital, Capital Medical University, Beijing, 100069 China; 2 Biomedical Information Center, Beijing Youan Hospital, Capital Medical University, Beijing, 100069 China; 3 Fourth Department of Liver Disease (Difficult & Complicated Liver Diseases and Artificial Liver Center), Beijing Youan Hospital, Capital Medical University, Beijing, 100069 China; 4 Beijing Municipal Key Laboratory of Liver Failure and Artificial Liver Treatment Research, Beijing, China

**Keywords:** Thermal ablation, Natural killer cells, Hepatocellular carcinoma

## Abstract

**Background:**

Thermal therapy induces an immune response in patients with hepatocellular carcinoma (HCC), but the dynamic characteristics of the natural killer (NK) cell immune response post-thermal ablation remain unclear. We conducted a prospective longitudinal cohort study to observe the dynamic changes of phenotype and function of NK cells in peripheral blood before and after thermal ablation of hepatitis B-associated HCC and their correlation with tumor recurrence.

**Methods:**

Fifty-six patients clinically and pathologically confirmed with hepatitis B-associated HCC were selected for thermal ablation. Peripheral blood was collected on day 0, day 7, and month 1. NK cell subsets, receptors, and killing function were detected by flow cytometry, and the LDH levels were examined. Overall recurrence and associated variables were estimated using Kaplan–Meier, log-rank, and Cox proportional-hazards analyses.

**Results:**

The frequency of CD3^−^CD56^+^ cells was increased on day 7 (*P* < 0.01) without significant differences between D0 and M1. NKG2D, NKp44, NKp30, CD159a, and CD158a expression was increased on M1 (all *P* < 0.05). The granzyme B and IFN-γ expression in NK cells were higher on M1 vs. D0 (*P* < 0.05). On day 7, the NK cell lysis activity of the target K562 cells was increased (*P* < 0.01) but decreased on M1 (*P* < 0.05). Survival analysis showed that CD158a expression and IFN-γ and perforin release on day 0 were associated with the risk of HCC recurrence. Cox regression analysis showed that the expression changes in CD56, NKp46, granzyme B, and perforin (D7-D0) induced by thermal ablation were associated with recurrence-free survival (RFS) of patients with HCC.

**Conclusion:**

Thermal ablation increased the frequency and function of CD3^−^CD56^+^ NK cells in the peripheral blood of patients with HCC. These cells tended to be more differentiated and activated. Notably, expression levels of NK cell receptors NKp46, perforin, and granzyme B were associated with RFS.

**Supplementary Information:**

The online version contains supplementary material available at 10.1186/s12885-023-10823-4.

## Introduction

Primary liver cancer is the most common malignant tumor of the liver, of which hepatocellular carcinoma (HCC) is the dominant type, accounting for more than 85%-90% of all cases. World cancer epidemiology studies suggest that HCC ranks sixth in tumor incidence and fourth in mortality, whereas HCC ranks third among all tumor-related deaths in China [1]. The main risk factors for liver cancer are HBV/HCV infection, alcoholism, non-alcoholic hepatitis, and diabetes [2]. Chronic HBV infection accounts for approximately 50% of liver cancer cases worldwide [3]. HCC treatment methods have been constantly updated and improved. In addition to the implementation of traditional hepatectomy, chemotherapy, radiotherapy, liver transplantation, transcatheter arterial chemoembolization, and ablation, the application of target drug therapy and immunotherapy has gradually increased in clinical practice, which has improved the survival rates of patients with advanced HCC [4].

Local ablative therapy is suitable for Chinese Liver Cancer (CNLC) stage 1a and part of stage 1b liver cancer patients (i.e., the presence of a single tumor with a diameter < 5 cm or of 2–3 tumors with a maximum diameter of < 3 cm), with no vascular, bile duct, and adjacent organ invasion or distant metastasis. Radical treatment effects can be obtained in Child–Pugh A-B liver function grading [5]. CLNC stage Ia includes patients Eastern Cooperative Oncology Group (ECOG) performance status (PS) 0–2, Child–Pugh A-B, single tumor ≤ 5 cm, no vascular invasion, and no metastases, while CLNC stage Ib includes patients ECOG PS 0–2, Child–Pugh A-B, a single tumor > 5 cm or 2–3 tumors ≤ 3 cm, no vascular invasion, and no metastases [5, 6]. The Child–Pugh score is calculated based on the presence of encephalopathy, presence of ascites, bilirubin levels, albumin levels, and prothrombin time [7]. In contrast to surgical resection, ablation has a more local treatment effect, achieving no significant difference in the 4-year overall survival (OS) and disease-free survival (DFS) between patients with HCC < 5 cm in diameter in previous studies [8, 9].

Thermal ablation of liver cancer includes radiofrequency ablation (RFA), microwave ablation (MWA), laser ablation, and ultrasound ablation, of which RFA and MWA are currently the most widely clinically used. Their principles are similar. Under the action of an electric field, polar molecules, and ions in the tumor tissue are induced to move violently and generate thermal energy. This effect leads to irreversible degeneration and histopathologic coagulative necrosis [10].

Currently, most of the published studies on the post-thermal ablation changes in the immune response of patients with HCC mainly focus on T-cell immunity. The roles of CD4^+^ T cells, CD8^+^ T cells, tumor-specific T cells [11, 12], central memory lymphocytes (CD45RA^−^/CCR7^+^) [12, 13], and infiltrating CD45RO^+^ memory T cells post-thermal ablation have been confirmed [11]. Higher tumor-specific CD8^+^ T cell counts are associated with increased survival in patients with HCC [14, 15]. In addition, enhanced CD45RO^+^ T cell infiltration is considered to be a marker of improved clinical outcomes of all types of solid tumors [16]. Moreover, RFA was established to decrease Treg levels [11, 17]. The subsequent increase in CD8 ^+^ T/Treg ratios indicates a shift in the immune system toward antitumor activities. These data are based on the results of a preclinical model investigation [18], but no large prospective randomized controlled trials have been conducted to explore the association between survival and ablation therapy in ablation-treated vs. untreated patients.

Currently, a few studies have been performed on the NK cell immune response of patients with HCC post-thermal ablation. Experimental data showed that hyperthermia enhanced the cytotoxic activity of NK cells, exerting an anti-proliferation effect on tumor cells. Significant differences were observed in relapse-free survival (RFS) between the two groups when assessed using cytotoxicity and/or enhancement of interferon-γ general increase in NK cell functional blood lymphocyte count and NK cell efficacy [13, 19]. Still, the etiology of liver cancer in the selected patients was mainly hepatitis C and alcoholic liver disease, the sample size was too small, and the sampling and time point matching were low.

Unlike B cells and T cells expressing a single antigen-specific receptor, NK cells express multiple activating and inhibitory receptors on their surface [20, 21]. NK cell inhibitory receptors have different molecular structures that can specifically bind to different class I molecular alleles. The high expression of KIR2DL1 (CD158a) and KIR2DL2/3 (CD158b) receptors, especially in patients expressing specific HLA-C ligands, is associated with tumor susceptibility but also with disease progression, with increased expression of these receptors in the NK cells of patients with advanced melanoma [22, 23]. Studies in patients with extensively metastatic melanoma, breast cancer, or non-small cell lung cancer (NSCLC) showed an increased expression of the CD158a and CD158b KIR receptors and a negative correlation with NK cell cytotoxicity [24, 25]. CD94/NKG2A inhibits NK cell activation [26]. The natural cytotoxic receptors NCRs are a group of NK cell-activated receptors, including NKp46, NKp30, and NKp44, that have antitumor immune activity in NCR ligand recognition of tumor cells [27]. NKG2D, as a key activating receptor, leads to the phosphorylation of DAP10, which activates mitogen-activated protein kinase (MAPK) and Janus kinase (Jak)/signal transductor and transcriptional activator (STAT) signaling pathways [28].

To the best of our knowledge, no reports exist on the dynamic changes in NK cell immunity in patients with HBV-associated HCC pre- and post-thermal ablation, as well as on the association of these phenotypic changes with cytokine secretion and cytotoxic capacity and tumor recurrence. In this study, we aimed to assess the immune dynamics of NK cells in the peripheral blood of patients with HCC pre- and post-thermal ablation and to establish their correlation with relapse-free survival (RFS) based on NK cell frequency, subgroup and receptor types, and function.

## Methods

### Ethical statement

The selected patients were admitted to Beijing Youan Hospital, Capital Medical University, from January 2020 to April 2021. They provided written informed consent for participation in the study. This study and other related experiments were approved by the Beijing Youan Hospital Research Ethics Committee (No. [2019]073). Informed written consent was obtained in accordance with the Declaration of Helsinki. The study was carried out in accordance with the approved guidelines and regulations.

### Study population

A prospective longitudinal queue design was used in the present investigation. The selected patients were admitted to Beijing Youan Hospital, Capital Medical University, from January 2020 to April 2021. The diagnosis of fifty-six patients with hepatitis B-related primary liver cancer (HCC) was clinically and pathologically confirmed based on the guidelines of the Primary Liver Cancer Diagnosis and Treatment Standards of the Medical Administration Authority of the National Health Commission of the People’s Republic of China (2019 edition). We included patients with a single tumor with a diameter of ≤ 3 cm in diameter or no more than two tumor nodules with the largest tumor diameter ≤ 3 cm, with no invasion in blood vessels, bile ducts, adjacent organs, or distant metastasis, and A Child–Pugh live r function grade. All patients combined with other virus infections, autoimmune conditions, or alcoholic liver disease were excluded. All included patients underwent thermal ablation (RFA or MWA) after admission without any immunotherapy within 6 months before enrollment. All subjects without ongoing co-infections or other diseases and their characteristics are summarized in Table 1.

**Table 1:** Characteristics of the study population (*n* = 56)

Baseline characteristics	Categories or median (range or %)	Number of patients
Sex	Male	44
	Female	12
Age (years)	61 (37–83)	
Child–pugh score	5	51
	6	5
TBIL (umol/L)	17.5 (6.1- 44.5)	
AIB (g/L)	39.8 (32.9–48.7)	
PLT (× 10^9^/L)	144 (55–316)	
PTA (%)	85 (67–108)	
Etiology (HBV)	HBV	56
Diameter of HCC (mm)	22.5 (9–40)	
AFP (ng/ml)	404 (1.39–17,694)	
BCLC staging	A	56
HBV-DNA (copies/ml)	≤ 10	30
	≤ 100	20
	≤ 1000	4
	≤ 10,000	2
HbeAg( +)/HbeAg(-)		11/45
Anti-viral therapy	ADV	2
	ETV	37
	TDF	5
	TAF	7
	ETV + ADV	1
	ETV + TDF	3
	LAM + ADV	1
Complete necrosis		56
No. of nodules	1	52
	2	4
Diagnosis	Clinical diagnosis	49
	Pathological diagnosis	7

*TBil* total serum bilirubin, *AIB* serum albumin, *PLT* platelet, *PTA* prothrombin activity, *AFP* α-fetoprotein, *HBV-DNA* hepatitis B virus DNA, *HBeAg* hepatitis Be antigen

### Study design

The selected patients underwent thermal ablation under conscious sedation and CT guidance. Three radiologists with 10 years of experience in interventional oncology performed the ablation. For RFA, we used the Cool-Tip RFA Electrode Kit (ACT1530) connected to a radiofrequency generator (Model 1500 Radiofrequency generator, RITA medical system, Inc., a Mountain View, CA). For MWA, we used ECO-100AL3 connected to a microwave generator (MTC-3C Nanjing Viking Jiuzhou Medical Device R&D Center). One month after ablation, an abdominal enhanced CT or MRI was performed for radiological evaluation. The efficacy of HCC treatment was determined according to the Evaluation Criteria of Solid Tumor Efficacy (mRECIST criteria). After complete ablation was confirmed, abdominal enhanced CT or MRI was performed every 3 months, and follow-up was performed until recurrence or study termination (November 1, 2021). The date of recurrence was determined by the first imaging study showing a clear recurrence. Imaging assessments were performed by radiologists with more than 10 years of experience during follow-up. For patients with recurrence, the endpoint was defined as the time point of recurrence, determined by imaging doctors according to imaging results. For patients with no recurrence, follow-up was censored on November 1, 2021.

Peripheral blood was collected before thermal ablation (D0), 1 week after thermal ablation (D7), and four weeks after thermal ablation (M1). NK cell subsets, receptors, and cell functions were determined by flow cytometry. NK cell activity was detected by the lactate dehydrogenase (LDH) release method. At M1, patients underwent CT or MRI scans to assess whether the thermal ablation was radical. After that, enhanced CT or MRI scans were performed every 3 months to assess possible disease recurrence. Thermal ablation and post-treatment evaluation were conducted by two interventional physicians (CXW and SB) with more than 10 years of clinical experience. For a single course of treatment for tumor ablation, a melting edge of 5–10 mm was the goal. At the end of the operation, the ablation area was observed by enhanced CT. If the edge was < 5 mm, the edge was expanded to ensure complete necrosis. The therapeutic efficacy of HCC met the efficacy evaluation criteria for solid tumors (m-RECIST standard) [29].

### Cell staining and flow cytometry analysis

**Fig. 1:**
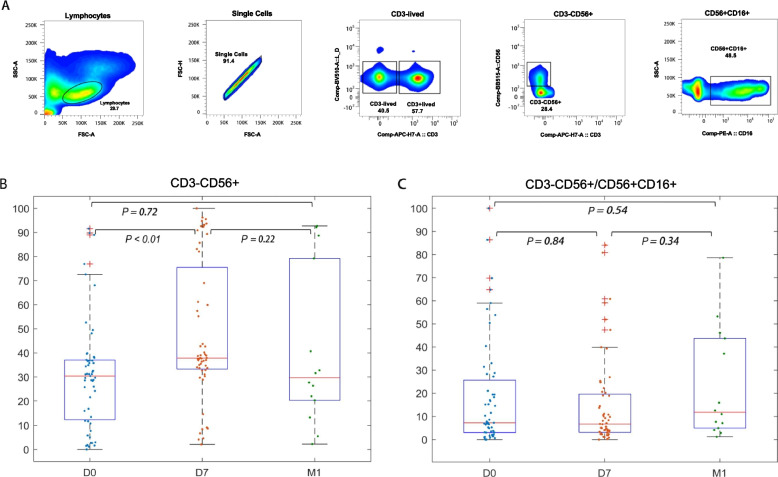
The NK cell ratio increased, mainly due to the significantly increased frequency of CD3^−^CD56^+^ cells. **A** The gating strategy used to identify NK cell subsets by flow cytometry and the frequencies of the gated populations are shown for a representative HC. The lymphocytes were gated according to forward and side scatter dot plots. Single cells were gated according to forward height and side scatters forward area. Dead cells were excluded by staining with Live/Dead fixable viability stain 510. NK cells were defined within the CD3^−^gate based on the expression of CD16 and CD56. **B** D0-D7, CD3^−^CD56 ^+^ cell frequency was significantly increased (*P* < 0.01). D7-M1, the frequency of CD3-CD56 + cells decreased (*P* = 0.22). **C** D0-M1, the frequency of CD3^−^CD56^+^CD16^+^ subsets was not significantly increased (*P* = 0.54)

**Fig. 2:**
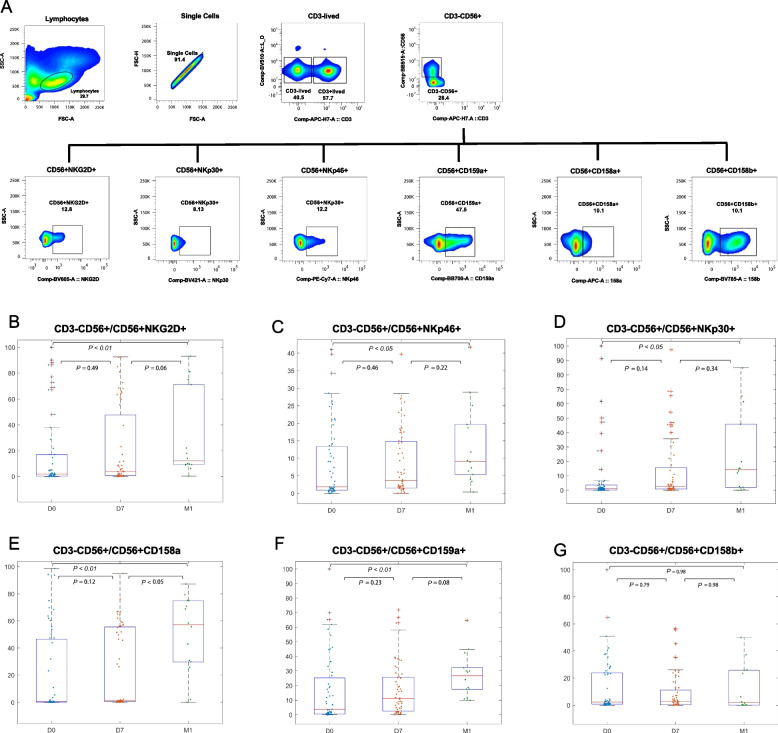
Thermal ablation cannot only increase the number of NK cells but also increase the expression of NK cell activating receptors and weaken the expression of NK cell inhibiting receptors. **B**, **C**, **D** D0-D7-M1 peripheral blood NK cell activation receptor (NKG2D, NKp44, NKp30) expression gradually increased, M1 peripheral blood NK cell activation receptor (NKG2D, NKp44, NKp30) expression significantly increased(*P* < 0.05), especially NKG2D (*P* < 0.01). **E**, **F** The frequency of D0-D7 inhibitory receptors NKG2A (CD159a) and CD158a did not change significantly but increased significantly after 4 weeks of thermal ablation (D0-M1) (*P* < 0.01). **G** The expression of CD158b in peripheral blood NK cells was not observed after thermal ablation. The Lilliefors test was used to test whether the data at the three time points conformed to the normal distribution and met the homogeneity of variance. For the data with a normal distribution and homogeneity of variance, ANOVA was used to analyze the differences in NK cell subsets, receptors, and functions at the three time points. Otherwise, the Kruskal–Wallis test was used to compare the changes in NK cell subsets, receptors, and functions at three time points before and after treatment. Tukey method was used for comparison between groups. *P* < 0.05 was a significant difference

**Fig. 3:**
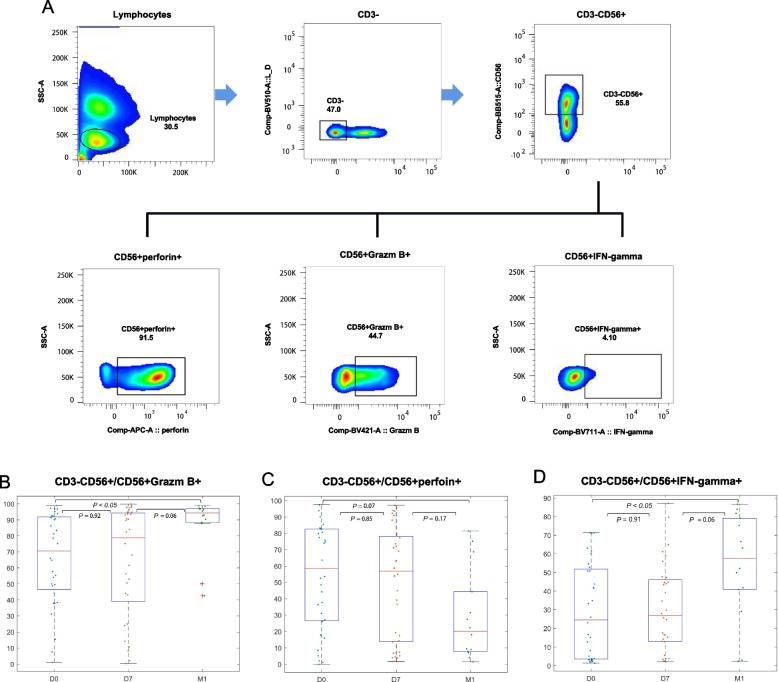
Thermal ablation enhanced NK cell lysis activity and cytokine release. **A** Cell surface CD3-APC-H7, CD56-BB515, and CD16-PE staining was performed first. After washing at 4℃ for 20 min, 2 mL of PBS was added, and the cells were centrifuged at 600 × g for 3 min to discard the supernatant. Then the cells were lysed using a membrane-breaking solution for 20 min at 4℃. After washing, IFN-γ-BV711, Perforin-AP647, Granzyme B-BV421, and CD107A-PE-CY7 were added and incubated at 4℃ for 20 min without light. **B** The expression of granzyme B was continuously increased in peripheral NK cells at 1 and 4 weeks after thermal ablation and significantly increased in peripheral NK cells at 4 weeks (*P* < 0.05). **C** The expression of perforin in peripheral blood NK cells was not increased at 1 week after thermal ablation but decreased at 4 weeks after thermal ablation (*P* = 0.07). **D** Increased IFN-γ production in peripheral NK cells was observed at weeks 1 and 4 after thermal ablation, and significantly increased IFN-γ production in peripheral NK cells was observed at week 4 (*P* < 0.05). The Lilliefors test was used to test whether the data at the three time points conformed to the normal distribution and met the homogeneity of variance. For the data with a normal distribution and homogeneity of variance, ANOVA was used to analyze the differences in NK cell subsets, receptors, and functions at the three time points. Otherwise, the Kruskal–Wallis test was used to compare the changes in NK cell subsets, receptors, and functions at three time points before and after treatment. Tukey method was used for comparison between groups. *P* < 0.05 was a significant difference

Peripheral blood mononuclear cells (PBMCs) were thawed in a water bath at 37 °C for 1 min, washed, and resuspended in Roswell Park Memorial Institute (RPMI) medium containing 10% v/v fetal calf serum. Antibodies were incubated at 4 °C for surface staining, and PBMCs were stained with the following fluorophore-conjugated human monoclonal antibodies at room temperature for 20 min: anti-CD3-APC-H7, anti-CD56-BB515, anti-CD16-PE (Fig. 1A), anti-NKp46-PE-cy7, anti-NKp30-BV421, anti-CD158a-APC, anti-CD158b-BV785, anti-NKG2D-BV605, and anti-CD159a-BB700 (Fig. 2A). For intracellular staining, the cells were permeabilized and further intracellularly stained with anti-IFN-γBV711, anti-Perforin-AP647, and anti-Granzyme B-BV421 (Fig. 3A). Cell viability was determined using Live/Dead fixable viability stain 510 (BD Biosciences, San Jose, CA, USA). The cells were washed and fixed with 2% paraformaldehyde. Cytometer setup and tracking calibration particles were used to ensure that fluorescence intensity measurements were consistent among all experiments. At least 200,000 PBMCs were acquired on a BD FACSCanto II flow cytometer. Gating on forward scatter and side scatter parameters was used to exclude cell debris from the analysis; the forward height and forward area were used to exclude doublets. Data analysis was performed using FlowJo 7.6.1 software version 10.4 (TreeStar, Ashland, OR, USA).

### Cytolytic assay

NK cell activity was detected by LDH release assay. The PBMCs were resuscitated, washed once, plated on a six-well plate, and incubated in a 37 °C incubator overnight. The cells were collected, 70-mesh filtered, centrifuged, counted, and adjusted at 100 µl containing 5 × 10^5^. K562 cells at 100 µl containing 5 × 10^3^ cells (effective target ratio of 100:1) were plated in 96-well plates and incubated at 37 °C overnight. Each specimen was tested in triplicate. The natural release control group and the maximum release control group of target cells were set simultaneously. Water (10 µl) was added to each natural release well, 10 µl lysate was added to each maximum release well, and the 37 °C incubation lasted 45 min. The supernatant (50 µl) was transferred to a new 96-well plate, 50 µl of the reaction solution was added (600 µl assay buffer and 11.4 mL of substrate stock solution) to each well, reacted at room temperature for 30 min, and stopped with 50 µl of stop solution. The plates were read at 490 nm. The percentage of cytotoxicity was calculated using the following formula: cytotoxicity (%) = (test sample—low control) / (high control—low control) %.

### Data analysis

After all the original data were verified, MATLAB 2018 software was used for statistical analysis. The Lilliefors test was used to assess whether the data at the three-time points conformed to the normal distribution and met the requirements for homogeneity of variance. For the data with normal distribution data and homogeneity of variance, ANOVA was used to analyze the differences in the NK cell subsets, receptors, and functions at three time points. Otherwise, the Kruskal–Wallis test was used to compare the changes in the NK cell subsets, receptors, and functions at the three time points pre- and post-treatment. Tukey’s post hoc test was used for inter-group comparisons. *P* < 0.05 was considered to indicate statistically significant differences.

RFS was calculated from the first ablation, and follow-up was calculated from the beginning of the ablation to the last follow-up. Based on the median data, the patients were divided into high-risk (high-recurrence) and low-risk (low-recurrence) groups, and their respective Kaplan–Meier curves were drawn. The Log-rank test was used to analyze the differences between the two survival curves. *P* < 0.05 was considered to indicate statistically significant differences.

Cox regression analysis was used to determine the factors associated with recurrence and to evaluate the correlation between the NK cell subsets, activity, and function changes in the peripheral blood of HCC patients pre- and post-thermal ablation and the recurrence-free survival of HCC patients. Cox regression was used to determine the variables of recurrence-free survival in multivariable analysis. *P* < 0.05 was considered to indicate statistically significant differences.

## Results

### Clinical outcome results

We recruited 56 patients with HBV-related HCC whose liver function was Child A and BCLN grade A stage. All patients were without ongoing co-infections or other diseases. Their characteristics are summarized in Table 1.

The median of each data at D0 was used to divide the patients into two groups. The clinical characteristics of the high-risk (*n* = 26) and low-risk (*n* = 25) groups are summarized in Tables 2,3,4, and 5. When grouped based on CD158a, compared with the low-risk group, the high-risk group had larger tumors (*P* = 0.019) (Table 2). When grouped based on NKG2D, there were no significant differences between the two groups (all *P* > 0.05) (Table 3). When grouped based on IFN-γ, compared with the low-risk group, the high-risk group had a lower prothrombin activity (*P* = 0.018) (Table 4). When grouped based on perforin, compared with the low-risk group, the high-risk group had lower platelets (*P* = 0.038) (Table 5).

**Table 2:** Clinical baseline characteristics of the patients in the high- and low-risk groups (CD158a)

Baseline characteristics	High-risk group (*n* = 26)	Low-risk group (*n* = 25)	*P*
Sex (male: female)	23:3	19:6	0.060
Age (years)	61 (46–77)	62 (37–83)	0.850
Child–Pugh (5:6)	24:2	23:2	0.631
TBIL (µmol/L)	18.7 (6.1–48.2)	17.3 (10.4–37.1)	0.523
AIB (g/L)	39.5 (33.6–47.6)	40 (32.9–48.7)	0.568
PLT (× 10^9^/L)	145 (55–316)	137 (61–232)	0.668
PTA (%)	83.5 (67–95)	86 (69–108)	0.213
Etiology (HBV)	HBV (100%)	HBV (100%)	> 0.999
Diameter of HCC (mm)	25.9 (9–40)	20 (9–39)	0.019
AFP (ng/ml)	88.7 (1.39–1096)	773.6 (1.69–17,694)	0.328
BCLC staging	A (100%)	A (100%)	> 0.999
HBV-DNA (copies/ml)	117.7 (10–2040)	372.3 (10–8015)	0.433

*TBil* total serum bilirubin, *AIB* serum albumin, *PLT* platelet, *PTA* prothrombin activity, *AFP* α-fetoprotein, *HBV-DNA* hepatitis B virus DNA

**Table 3:** Clinical baseline characteristics of the patients in the high- and low-risk groups (NKG2D)

Baseline characteristics	High-risk group (*n* = 26)	Low-risk group (*n* = 25)	*P*
Sex (male: female)	22:4	20:5	0.713
Age (years)	62 (46–77)	65 (56–71)	0.884
Child–Pugh (5:6)	22:3	23:2	> 0.999
TBIL (µmol/L)	18.8 (8.9–48.2)	16.1 (6.1–26.5)	0.332
AIB (g/L)	40.6 (34.3–47.6)	40 (33.6–48.7)	0.554
PLT (× 10^9^/L)	131 (55–248)	140 (70–232)	0.421
PTA (%)	82 (67–94)	86 (76–104)	0.317
Etiology (HBV)	HBV(100%)	HBV (100%)	> 0.999
Diameter of HCC (mm)	25.8 (17–36)	22.5 (9–40)	0.174
AFP (ng/ml)	798.5 (1.39–17,694)	67.1 (2.05–818.0)	0.306
BCLC staging	A (100%)	A (100%)	> 0.999
HBV-DNA (copies/ml)	359.7 (10–8015)	131.0 (10–2040)	0.490

*TBil* total serum bilirubin, *AIB* serum albumin, *PLT* platelet, *PTA* prothrombin activity, *AFP* α-fetoprotein, *HBV-DNA* hepatitis B virus DNA

**Table 4:** Clinical characteristics of baseline patients in the high-risk and low-risk groups (IFN-γ)

Baseline characteristics	High-risk group (*n* = 16)	Low-risk group (*n* = 15)	*P*
Sex (male: female)	12:4	12:3	0.739
Age (years)	59 (44–74)	61 (37–83)	0.579
Child–pugh (5:6)	15:1	13:2	0.953
TBIL (µmol/L)	16.3 (8.6–26.5)	17.3 (6.1–37.1)	0.540
AIB (g/L)	39.2 (33.6–42.8)	40.1 (32.9–48.7)	0.477
PLT (× 10^9^/L)	139.9 (59–316)	153.4 (80–232)	0.332
PTA (%)	83 (71–95)	91.6 (69–108)	0.018
Etiology (HBV)	HBV (100%)	HBV (100%)	> 0.999
Diameter of HCC (mm)	24 (9–40)	18 (9–39)	0.477
AFP (ng/ml)	69.0 (2.05–818)	1196.2 (1.55–17,694)	0.331
BCLC staging	A (100%)	A (100%)	> 0.999
HBV-DNA (copies/ml)	670.9 (10–8015)	52.3 (10–253)	0.246

*IFN-γ* interferon-γ, *TBil* total serum bilirubin, *AIB* serum albumin, *PLT* platelet, *PTA* prothrombin activity, *AFP* α-fetoprotein, *HBV-DNA* hepatitis B virus DNA, *HBeAg* hepatitis Be antigen

**Table 5:** Clinical characteristics of baseline patients in the high-risk group and low-risk group (perforin)

Baseline characteristics	High-risk group (*n* = 15)	Low-risk group (*n* = 16)	*P*
Sex (male: female)	11:4	13:3	0.598
Age (years)	62 (47–83)	59 (37–71)	0.634
Child–pugh (5:6)	13:2	14:2	0.641
TBIL (µmol/L)	17.4 (8.6–26.5)	16.1 (6.1–37.1)	0.453
AIB (g/L)	39 (33.6–44.5)	40.1 (32.9–48.7)	0.384
PLT (× 10^9^/L)	128 (59–319)	163 (80–232)	0.038
PTA (%)	86.7 (71–104)	87.6 (69–108)	0.937
Etiology (HBV)	HBV (100%)	HBV (100%)	> 0.999
Diameter of HCC (mm)	22.7 (9–40)	19.5 (9–39)	0.302
AFP (ng/ml)	65.0 (2.33–818)	1129.5 (1.55–17,694)	0.359
BCLC staging	A (100%)	A (100%)	> 0.999
HBV-DNA (copies/ml)	709.0 (10–8015)	55.3 (10–253)	0.220

*TBil* total serum bilirubin, *AIB* serum albumin, *PLT* platelet, *PTA* prothrombin activity, *AFP* α-fetoprotein, *HBV-DNA* hepatitis B virus DNA

### Frequency of circulating NK cells increased post-thermal ablation

First, we aimed to determine the effect of thermal ablation on the frequency and subsets of NK cells in the peripheral blood of HCC patients. In order to achieve this purpose, we used CD3, CD56, and CD16 antibodies for NK cell identification and classification. Based on the results, we divided the NK cells into two subgroups: CD3^−^CD56^bright^ and CD3^−^CD56^dim^CD16^+^. At D0-D7, the frequency of CD3^−^CD56^+^ cells was increased (*P* < 0.01) (Fig. 1B), while the frequency of CD3^−^CD56^dim^CD16^+^ cells was not significantly increased (*P* = 0.84) (Fig. 1C). At D7-M1, the frequency of CD3^−^CD56^+^ cells (Fig. 1B) and CD3^−^CD56^dim^CD16^+^ subsets were not significantly different (*P* = 0.22 and *P* = 0.54) (Fig. 1C). Therefore, the early increase in NK cell frequency post-thermal ablation was mainly caused by a significant rise in the frequency of CD3^−^CD56^+^ cells, whereas the more potent CD3^−^CD56^dim^CD16^+^ subgroup was dominant at 4 weeks (M1) post-ablation.

### NK cell receptor expression was induced by thermal ablation

NK cell activity is closely regulated by many activating and inhibiting receptors, whose balance determines the final activity and function of NK cells. In order to investigate the effect of ablation on NK cell activation, flow cytometry was used to analyze the expression changes in activating (NKG2D, NKp46, and NKp30) and inhibitory (NKG2A, CD158a, and CD158b) receptors on the NK cell surface before ablation (D0), 1 week after ablation (D7), and 4 weeks after ablation (M1). The results showed that the expression levels of the activating receptors (NKG2D, NKp46, and NKp30) in the peripheral blood of HCC patients were not significantly different between D0 and D7 but were increased from D0 to M1 (all *P* < 0.05) (Fig. 2B-D). The elevation of NKG2D was the most significant (*P* < 0.01). The expression of the inhibitory receptors on the surface of NK cells (CD158a and CD159a) were not significantly different on D7 (all *P* > 0.05) but were increased from D0 to M1 (*P* < 0.01) (Fig. 2 E, F). The expression of CD158b did not change significantly (Fig. 2G).

### Thermal ablation enhanced NK cell lysis activity and cytokine release

NK cell activation induces the release of cytokines and is involved in cytolysis. In order to further clarify the effect of NK cell activation by thermal ablation on its function, we compared the pre- and post-thermal ablation NK cell lysis activity and cytokine release in the peripheral blood of patients with HCC. The results showed that the expression of granzyme B in NK cells increased was not significantly different on D7 but was increased on M1 (*P* < 0.05) (Fig. 3B). The expression of perforin did not change significantly (Fig. 3C). The expression of IFN-γ, a representative cytokine released by NK cells, was not changed on D7 but was increased on M1 (*P* < 0.05) (Fig. 3D). These results suggested that granzyme B and IFN-γ increased and perforin decreased one month after thermal ablation.

### Thermal ablation enhanced the lysis and killing activity of NK cells

**Fig. 4:**
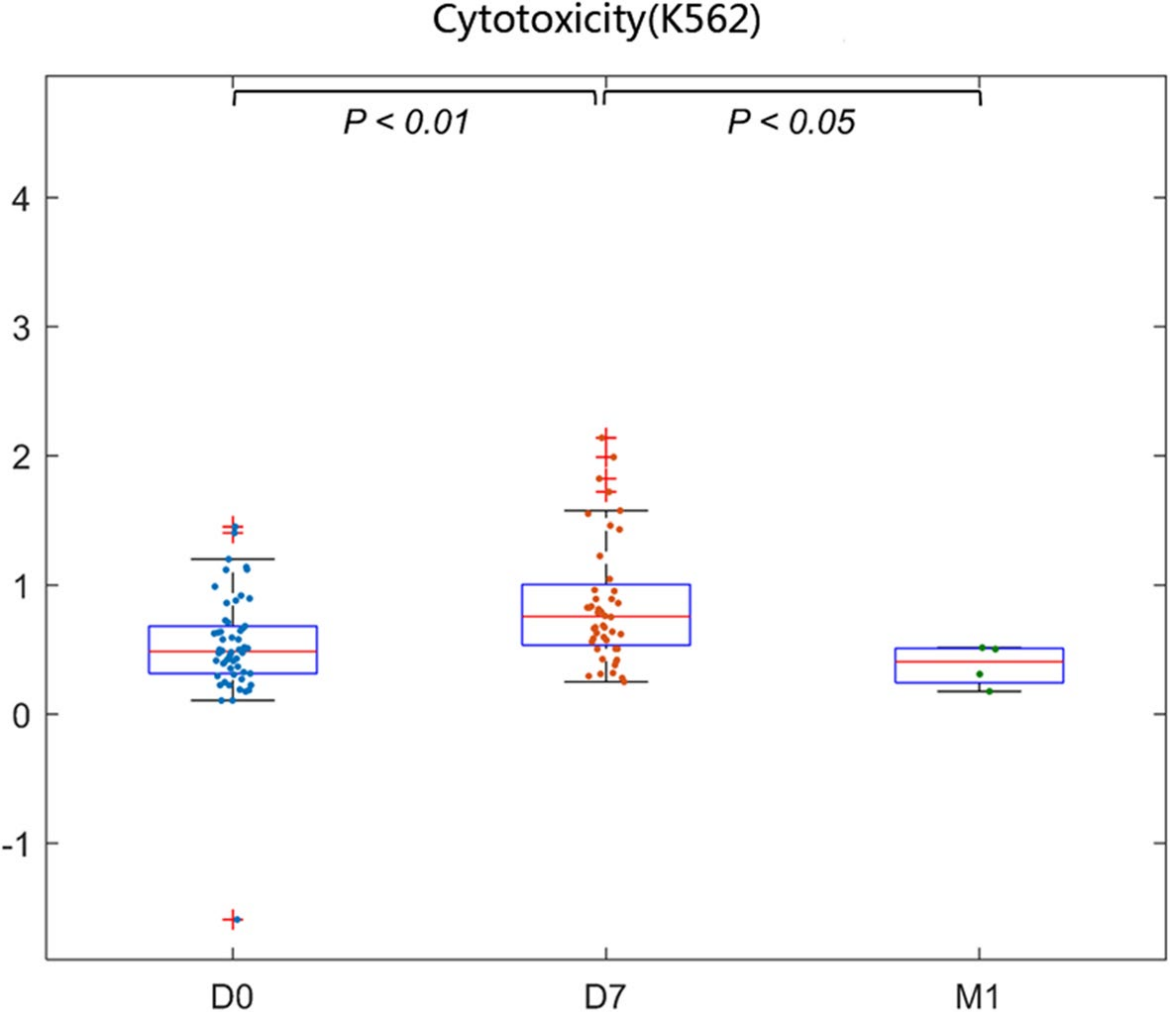
NK cell activity was detected by the lactate dehydrogenase (LDH) release method, suggesting that the activity of NK cell lysis in the peripheral blood of patients with primary liver cancer was significantly increased 1 week after thermal ablation (*P* < 0.01). At 4 weeks after thermal ablation, the cytotoxicity of peripheral NK cells to target K562 cells was significantly reduced (*P* < 0.05), and there were no significant differences in cytotoxicity of peripheral NK cells to target K562 cells between D0 and M1 patients with primary liver cancer

In addition, we also analyzed the effects of thermal ablation on NK cell lysis and killing activity in the peripheral blood of HCC patients. The killing activity of NK cells was detected using the lactate dehydrogenase (LDH) release assay. The cytotoxic activity of peripheral blood NK cells on target K562 cells was significantly increased on D7 (*P* < 0.01) and then decreased on (M1) (*P* < 0.05) (Fig. 4). Therefore, the lysis and killing activity of NK cells in the peripheral blood of patients with HCC after thermal ablation showed an initial increasing trend, followed by a decreasing one.

### NK cell dynamics were associated with RFS

After a median follow-up of 290 days (49–664) after ablation, 26 had a tumor recurrence. The median RFS was 384 days. In the univariable Cox regression analyses of the variables potentially associated with HCC recurrence (Table 6), CD3^−^ CD56^+^ NK cells (HR = 0.97, 95% CI: 0.95–0.99, *P* = 0.01) at D0 were significantly associated with tumor recurrence. In the multivariable analysis, variation of the CD3^−^ CD56^+^ NK cells (HR = 0.98, 95% CI: 0.96–1.00, *P* = 0.04) was independently associated with tumor recurrence.

**Table 6:** In a Cox regression analysis of variables potentially associated with HCC recurrence

	Univariable analysis			Multivariable analysis		
Variables	HR	95% CI	*P*	HR	95% CI	*P*
Age	0.99	0.96–1.03	0.78	0.98	0.94–1.03	0.42
Sex	1.75	0.60–5.12	0.31	1.41	0.43–4.65	0.57
HBV-DNA	1.17	0.53–2.57	0.69	1.18	0.51–2.72	0.70
HCC size	1.04	0.99–1.08	0.09	1.04	0.99–1.09	0.13
HCC no	2.00	0.46–8.70	0.35	0.86	0.17–4.44	0.86
AFP	1.00	1.00–1.00	0.44	1.00	1.00–1.00	0.94
CD3^−^CD56^+^	0.97	0.95–0.99	0.01	0.98	0.96–1.00	0.02

*HR* hazard ratio, *CI* confidence interval, *AFP* α-fetoprotein, *HBV-DNA* hepatitis B virus DNA, *HCC* hepatocellular carcinoma

**Fig. 5:**
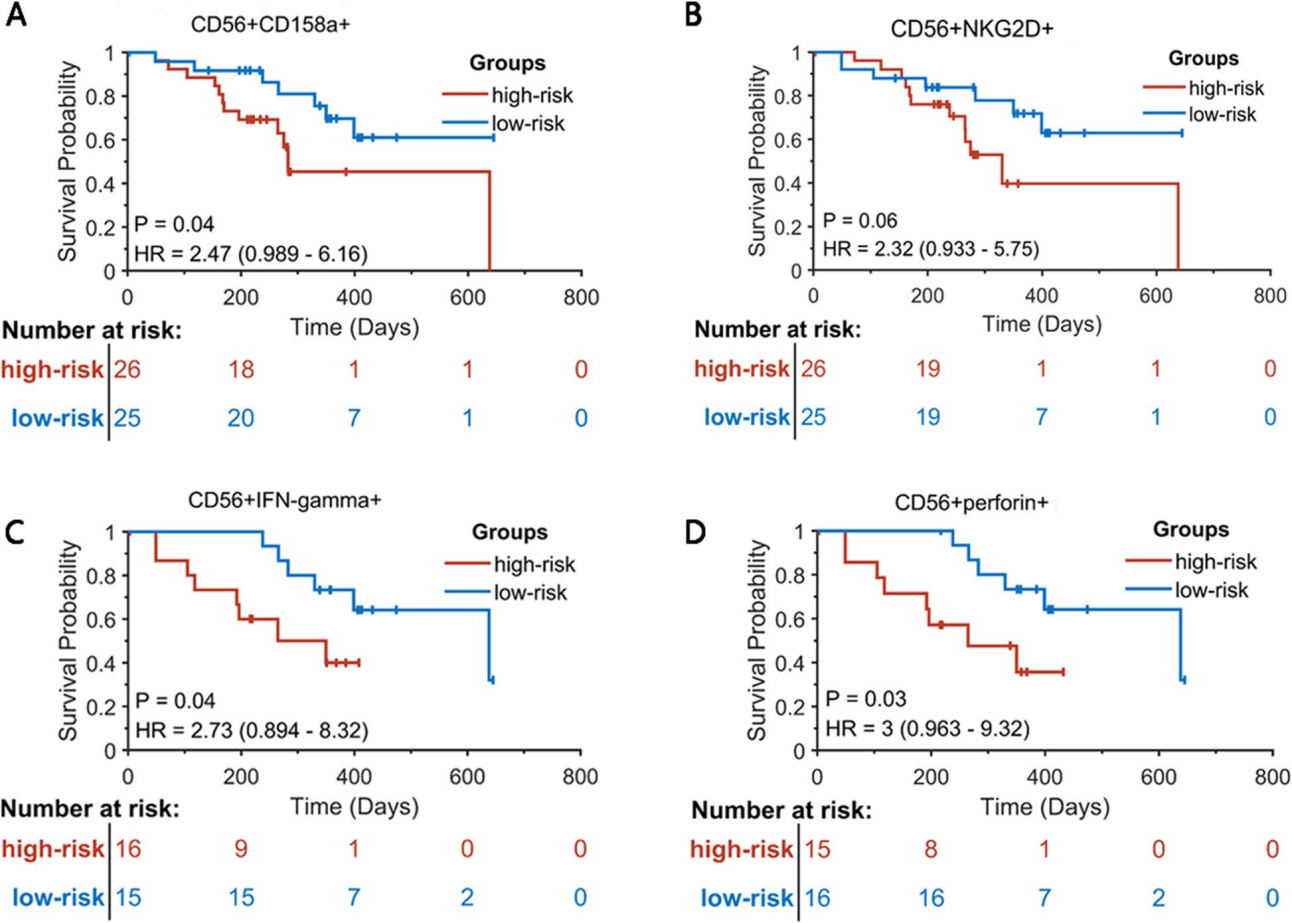
Patients with HCC (*n* = 56) were divided into the high-risk and low-risk groups according to the median NK cell subsets, activity, and function data on D0 (see Tables 1, 2, 3 and 4 for detailed clinical data). The KM curves were drawn to screen the indicators related to patients’ recurrence risk. CD158a expression (HR = 2.47, *P* = 0.0398) **A**, IFN-R (HR = 2.73, *P* = 0.0419) **C**, and perforin (HR = 3, *P* = 0.026) **D** were associated with the risk of HCC recurrence. NKG2D (HR = 2.32, *P* = 0.06) **B**

We investigated the associations between the NK cell subsets, activity, and function in the peripheral blood of patients with HCC before and post-thermal ablation and the recurrence-free survival of patients with HCC. The 56 patients with HCC were divided into high-risk and low-risk groups according to the median NK cell subsets, activity, and function data on D0, and the K-M curves were drawn to screen indicators related to the recurrence risk. CD158a expression (HR = 2.47, *P* = 0.0398) (Fig. 5A), IFN-γ (HR = 2.73, *P* = 0.0419) (Fig. 5C), and perforin (HR = 3.00, *P* = 0.026) (Fig. 5D) were associated with the risk of HCC recurrence, but not NKG2D (HR = 2.32, *P* = 0.06) (Fig. 5B).

**Fig. 6:**
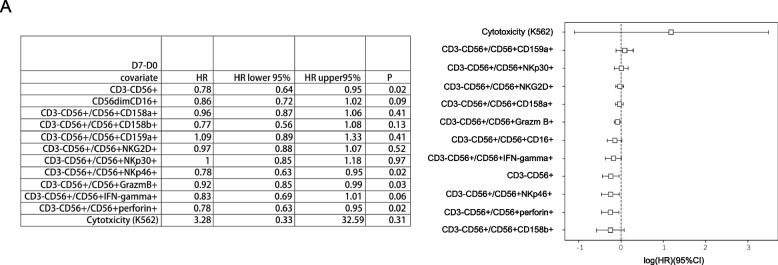
Cox regression analysis was used to evaluate the correlation between the NK cell subsets, activity, and function changes in peripheral blood of HCC patients before and after thermal ablation (D0 and D7) and the recurrence-free survival of HCC patients. D7-D0: CD56 (HR = 0.78, 95%CI: 0.64–0.95, *P* = 0.02), NKp46 (HR = 0.78, 95%CI: 0.63–0.95, *P* = 0.02), granzyme B (HR = 0.92, 95%CI: 0.85–0.99, *P* = 0.03), and perforin (HR = 0.78, 95%CI: 0.63–0.95, *P* = 0.02) were correlated with the postoperative recurrence-free survival of HCC patients

The Cox regression analysis was used to evaluate the correlation between the NK cell subsets, activity, and function changes in the peripheral blood of patients with HCC before and after thermal ablation (D7-D0) and the RFS of patients with HCC. CD56 (HR = 0.78, 95%CI: 0.64–0.95, *P* = 0.02), NKp46 (HR = 0.78, 95%CI: 0.63–0.95, *P* = 0.02), granzyme B (HR = 0.92, 95%CI: 0.85–0.99, *P* = 0.03), and perforin (HR = 0.78, 95%CI: 0.63–0.95, *P* = 0.02) were correlated with postoperative RFS of patients with HCC (Fig. 6).

## Discussion

In this study, we investigated the dynamic changes in peripheral blood NK cell frequency, activity, and function before and after thermal ablation of HBV-associated HCC and analyzed the correlation between the changes in the related indexes and HCC recurrence. The frequency of circulating NK cells was increased after ablation, and CD3^−^CD56 ^+^ cells were mainly increased on D7, whereas CD3^−^CD56^+^CD16^+^ cells were unchanged throughout M1. Three activating receptors (NKG2D, NKp46, and NKp30) were significantly increased on M1, and two inhibitory receptors (CD158a and CD159a) were significantly increased on M1, whereas the expression of the inhibitory receptor CD158b was not changed after thermal ablation. Thermal ablation increased the cytolytic activity of NK cells and cytokine release in the peripheral blood of patients with HCC, including granzyme B and IFN-γ, but perforin. Ablation initially increased the killing activity of NK cells in the peripheral blood of patients with HCC, but it then decreased. The Kaplan–Meier survival curve and Cox regression analyses suggested post–pre differences in CD3^−^CD56^+^, CD158a, CD158b, NKp46, granzyme B, perforin, and IFN-γ were associated with RFS in patients with HCC.

NK cells belong to the lymphocyte family [29], accounting for 10%-20% of all peripheral blood mononuclear cells (PBMC). NK cells are the body’s first line of defense against cancer cells and viral infection and can directly and nonspecifically kill tumor cells. This natural killing activity requires neither antigen sensitization nor antibody participation and has no MHC limitation. In addition to the powerful killing function, a strong immune regulation function has been established and interacts with the activities of other types of immune cells in the body to regulate the immune state and immune function of the body. Mature NK cells are derived mainly from NK progenitor cells. After five successive stages, CD56^bright^ NK cells differentiate into mature cytotoxic CD56^dim^CD16^+^ NK cells at “stage IV” in the bone marrow or peripheral blood. CD56^dim^CD16^+^ cells are rich in perforin and granzyme particles and have a strong killing function, and the CD56^bright^CD16 low-density or deficient subpopulation produces abundant cytokines (interferon γ, TNF, IL-10, IL-13, and GM-CSF) [30]. Our study showed that the frequency of CD3^−^CD56^+^ cells was significantly increased 1 week after ablation, whereas the frequency of the CD3^−^CD56^+^CD16^+^ subset was not. The early increase in NK cell frequency after ablation might be caused by the significantly increased frequency of CD3^−^CD56^bright^ cells. Four weeks after ablation, the frequency of CD3^−^CD56^+^ cells decreased to the level of D0, while CD3^−^CD56^dim^CD16^+^ cells remained unchanged, suggesting that thermal ablation can promote the maturation of NK cells and improve the killing function.

NK cell activation occurs in malignant transformed cells that have lost MHC class I molecules and are susceptible to lysis. Unlike adaptive T and B lymphocytes, NK cells do not rearrange their receptor genes but rely on a fixed number of NK cell receptors that inhibit and activate their respective ligands. NK cell degranulation occurs after immune synapse formation between NK cells and target cells and when activation-induced signals exceed a certain threshold, causing the death of the target cells. The cytotoxic particles of NK cells are composed mainly of perforin and granzyme proteins. Perforin exists constitutionally in NK cells and forms transmembrane pores on the surface of the target cells, leading to osmotic lysis [31, 32]. Perforin can lead to the gradual death of target cells by osmotic dissolution. On the other hand, in cells that have successfully avoided perforin-induced osmotic lysis, e.g., by the endocytosis of cells inserted into the membrane region of perforin-granzyme, it is released to induce apoptosis that kills those cells. In fact, perforin seems to activate clathrin and dynamin-dependent endocytosis to some extent. In addition, cytotoxic cells can attack tumors or cells invaded and integrated by viruses through various mechanisms, such as tumor necrosis factor-induced killing and Fas-FasL-induced apoptosis. The perforin/granzyme pathway is the only one of these that may cause necrosis [33].

NK cells can also produce cytokines. IFN-γ can induce non-apoptotic cell death by inducing autophagy in HCC. IFN-γ is a multipotent cytokine, from chronic inflammatory cell transformation, monocytes/macrophages, to antitumor, cytotoxic T cells (CTL), Th1, NK, and NKT cells, which can have opposite effects on host cells during an immune response. IFN-γ can directly or indirectly induce tumor cell apoptosis by up-regulating the expression of FAS and DR5. This cytokine can induce cell cycle arrest and establish tumor cell dormancy [34].

Immune regulation was induced by percutaneous thermal ablation in patients with HCC. In the case of RFA and MWA, heat transferred to the cancer tissue triggers cell death through a process called necrosis, which leads to the uncontrolled release of cell components into the extracellular space, triggering an inflammatory response. Initially, innate immune cells respond rapidly and infiltrate tissue sites, subsequently activating adaptive immune cells [11, 35]. This process also triggers an antitumor response because these tumor-specific antigens are exposed to components of the entire immune system. Locally applied therapy can trigger a distant antitumor response, a phenomenon known in radiotherapy as the Abscopal effect [36]. This phenomenon does not exist in traditional surgical resection because surgical resection involves direct removal of the tumor rather than local destruction.

According to our data, the frequency of CD3^−^CD56^+^ cells was significantly increased at D0-D7, but this frequency decreased at D7-M1. NK cell activity was detected by the LDH release method, suggesting that the lysis activity of NK cells on PBMCs from patients with HCC was significantly increased 1 week after ablation. Four weeks after thermal ablation, the cytotoxicity of peripheral NK cells to target cell K562 was significantly reduced. There were no significant differences in cytotoxicity of peripheral NK cells to K562 cells between D0 and M1 for patients with HCC. The cytotoxic effect was strongest one week after ablation and persisted for 1 month.

The activation of activating receptors (NKG2D, NKp46, and NKp30) and inhibitory receptors (NKG2A and CD158a) peaked at M1. The expression of inhibitory receptor CD158b did not change significantly after ablation. The “dynamic equilibrium” of NK cells is regulated by various receptor proteins on the cell surface. Even in the presence of inhibitory signals, tumor cells can also bind to KAR through overexpression of surface antigens and express inducible stress ligands by activating NK cell receptors. At this point, the activation signal exceeds the inhibitory signal, and NK cells are activated to kill target cells. The data showed that thermal ablation promoted NK cells to produce more activation signals than inhibition signals, which then activated NK cells to kill target cells. The changes in the NK cells induced by thermal ablation mainly occurred during D0-D7 and disappeared after 1 month. The activation of the activating receptors and inhibition receptors peaked at 1 month. The change of frequency and function of NK cells mainly occurred during D0-D7. Therefore, the lysis and killing activity of NK cells on PBMCs from HCC patients showed a trend of rising first and then falling post-thermal ablation. The increase in the frequency and function of NK cells occurred during D0-D7, so we evaluated the RFS according to D0 and differences in D0-7.

Overall, this study has several limitations. First, the sample size of M1 was small. Due to COVID-19, many people could be followed up only locally, resulting in a decrease in the sample size of M1. Second, since only blood was used, it was impossible to determine whether the observed changes were due to tumor invasion or hemodynamics. Third, cryopreservation has been described as influencing the viability of bone marrow cells (especially granulocytes that we did not study), but for practical reasons, it was not possible to handle fresh blood samples in this study.

In summary, our data suggest that thermal ablation induces NK cells to produce more activation signals than inhibition signals, activating NK cells to kill target cells. The activity of peripheral NK cell lysis and killing target K562 cells is significantly enhanced after thermal ablation at one week. Thermal ablation induces NK cell lysis and increases killing activity, lasting about 4 weeks. Thermal ablation induces NK cell expansion and activation, providing a beneficial antitumor effect and is associated with RFS. Our data suggest that the inhibitory (CD158a) and activating NKP46 receptors are related to the RFS, and it can be hypothesized that the activated receptor NKp46 plays a significant role in the killing effect of NK cells activated by thermal ablation. This change is based on the biodynamics induced by thermal ablation, which maybe provides a further direction for targeted immunity [37].

## Supplementary Information

**Additional file 1.** 

**Additional file 2.** 

**Additional file 3.** 

## Data Availability

The datasets used and/or analyzed during the current study are available from the corresponding author upon reasonable request.
