# Supplementary material for: Dynamic changes of phenotype and function of natural killer cells in peripheral blood before and after thermal ablation of hepatitis B associated hepatocellular carcinoma and their correlation with tumor recurrence
Source: BMC Cancer. 2023 May 30;23:486. doi: 10.1186/s12885-023-10823-4 (PMC10228897; doi:10.1186/s12885-023-10823-4)
Supplement: Supplementary file 1 — Additional file 1. [file 12885_2023_10823_MOESM1_ESM.docx]

Characteristics of patient clinical data

| Patient no | Gender | Age (y) | Etiology | No. of nodules | Cumulative diameter (mm) | Previous treatments | Complete necrosis | Phenotypic characterization D0 | Intracellular cytokine staining D0 | Cytotoxicity (K562) D0 | Phenotypic characterization D7 | Intracellular cytokine staining D7 | Cytotoxicity (K562)D7 | Phenotypic characterization M1 | Intracellular cytokine staining M1 | Cytotoxicity (K562) M1 | The operation type | follow-up time(day) | recurrence | AFP（ng/ml）D0 | HBV(HBeAg/HBeAb) | HBV-DNA（copies/ml) | pathology | antiviral therapy | FIB4 | Child-Pugh |
| --- | --- | --- | --- | --- | --- | --- | --- | --- | --- | --- | --- | --- | --- | --- | --- | --- | --- | --- | --- | --- | --- | --- | --- | --- | --- | --- |
| 1 | male | 57 | HBV | 1 | 35 | no | yes | √ |  | √ | √ |  | √ |  |  | √ | RFA | 161 | yes | 754 | HBeAb+ | 《10 | no | ETV | 0.2 | 5 |
| 2 | male | 55 | HBV | 1 | 23 | yes | yes | √ |  | √ | √ |  | √ |  |  |  | RFA | 241 | no | 57 | HBeAb+ | 《10 | no | ADV | 0.1 | 5 |
| 3 | male | 74 | HBV | 1 | 25 | yes | yes | √ | √ |  | √ | √ | √ |  |  |  | RFA | 387 | no | 4.54 | HBeAb+ | 《10 | yes | ETV | 0.7 | 5 |
| 4 | female | 66 | HBV | 1 | 20 | no | yes | √ | √ | √ | √ | √ | √ | √ | √ | √ | RFA | 664 | no | 5.63 | HBeAb+ | 《10 | no | ETV | 0.3 | 5 |
| 5 | female | 47 | HBV | 1 | 36 | no | yes | √ | √ | √ | √ | √ |  |  |  |  | RFA | 265 | yes | 52.7 | HBeAg+ | 《100 | no | ETV | 0.3 | 6 |
| 6 | male | 71 | HBV | 1 | 10 | yes | yes | √ |  | √ | √ |  | √ |  |  |  | RFA | 227 | no | 2.07 | HBeAb+ | 《10 | no | ADV | 0.6 | 5 |
| 7 | male | 63 | HBV | 1 | 21 | yes | yes | √ | √ | √ | √ | √ | √ |  |  |  | RFA | 451 | no | 9.28 | HBeAb+ | 《10 | no | TAF | 0.3 | 5 |
| 8 | male | 58 | HBV | 1 | 17 | no | yes | √ | √ | √ | √ | √ |  |  |  |  | RFA | 387 | no | 2.91 | HBeAb+ | 《10 | no | TDF | 0.1 | 5 |
| 9 | female | 70 | HBV | 2 | 33 | no | yes | √ | √ | √ | √ | √ | √ |  |  |  | RFA | 235 | no | 3.11 | HBeAb+ | 《100 | no | ETV | 0.7 | 5 |
| 10 | male | 58 | HBV | 1 | 30 | no | yes | √ |  | √ | √ |  | √ |  |  |  | RFA | 275 | yes | 5.74 | HBeAb+ | 《10 | no | ETV | 0.2 | 5 |
| 11 | female | 66 | HBV | 1 | 18 | no | yes | √ |  | √ | √ |  | √ |  |  |  | RFA | 306 | no | 4.22 | HBeAb+ | 《100 | no | ETV | 0.3 | 5 |
| 12 | male | 62 | HBV | 1 | 30 | no | yes | √ |  | √ | √ |  | √ |  |  |  | RFA | 298 | no | 8.02 | HBeAb+ | 《100 | no | TAF | 0.1 | 5 |
| 13 | male | 68 | HBV | 1 | 30 | yes | yes | √ |  | √ | √ |  | √ |  |  | √ | RFA | 299 | no | 1.39 | HBeAb+ | 《10 | no | ETV | 0.6 | 5 |
| 14 | male | 47 | HBV | 1 | 20 | no | yes | √ |  | √ | √ |  | √ |  |  |  | RFA | 297 | no | 421 | HBeAb+ | 《10 | no | TAF | 0.3 | 5 |
| 15 | male | 56 | HBV | 1 | 9 | yes | yes | √ | √ | √ | √ | √ | √ |  |  |  | RFA | 49 | yes | 2.68 | HBeAb+ | 《100 | no | ETV | 0.3 | 5 |
| 16 | female | 68 | HBV | 1 | 26 | yes | yes | √ |  | √ | √ |  | √ |  |  |  | RFA | 170 | yes | 44.4 | HBeAb+ | 《10 | yes | ETV+ADV | 0.8 | 6 |
| 17 | male | 61 | HBV | 1 | 12 | no | yes | √ | √ | √ | √ | √ | √ | √ | √ |  | RFA | 330 | yes | 2 | HBeAb+ | 《10 | no | ETV | 0.2 | 6 |
| 18 | male | 54 | HBV | 2 | 32 | yes | yes | √ | √ | √ | √ | √ | √ |  |  |  | RFA | 234 | no | 34 | HBeAb+ | 《10 | no | ETV | 0.1 | 5 |
| 19 | female | 46 | HBV | 1 | 18 | no | yes | √ |  | √ | √ |  | √ |  |  |  | RFA | 242 | no | 20.1 | HBeAb+ | 3.83*10*3 | no | ETV | 0.2 | 5 |
| 20 | male | 66 | HBV | 1 | 17 | yes | yes | √ | √ | √ | √ | √ | √ | √ | √ |  | RFA | 196 | yes | 10.3 | HBeAg+ | 2.04*10*3 | no | ETV | 0.4 | 5 |
| 21 | male | 63 | HBV | 1 | 20 | no | yes | √ |  | √ | √ |  | √ |  |  |  | RFA | 313 | no | 12 | HBeAb+ | 《10 | no | TAF | 0.1 | 5 |
| 22 | male | 55 | HBV | 1 | 24 | no | yes | √ |  | √ | √ |  | √ |  |  |  | RFA | 154 | yes | 6.93 | HBeAb+ | 《10 | no | ETV | 0.1 | 5 |
| 23 | male | 59 | HBV | 1 | 19 | yes | yes | √ |  | √ | √ |  | √ |  |  |  | RFA | 294 | yes | 3.11 | HBeAb+ | 《10 | no | ETV | 0.4 | 5 |
| 24 | male | 60 | HBV | 1 | 14 | no | yes | √ |  | √ | √ |  | √ |  |  |  | RFA | 228 | no | 104 | HBeAb+ | 《100 | no | ETV | 0.6 | 5 |
| 25 | female | 52 | HBV | 1 | 9 | no | yes | √ |  | √ | √ |  | √ |  |  |  | RFA | 221 | no | 149 | HBeAg+ | 1.95*10* | no | ETV+TDF | 0.3 | 5 |
| 26 | female | 61 | HBV | 1 | 10 | yes | yes | √ | √ | √ | √ | √ | √ |  |  |  | RFA | 430 | no | 2.55 | HBeAb+ | 《100 | no | ETV | 0.6 | 6 |
| 27 | male | 67 | HBV | 1 | 40 | yes | yes | √ |  | √ | √ |  | √ |  |  |  | RFA | 297 | no | 6.06 | HBeAb+ | 《10 | no | ETV | 0.2 | 5 |
| 28 | male | 66 | HBV | 1 | 22 | no | yes | √ | √ | √ | √ | √ |  | √ | √ |  | RFA | 402 | no | 2.05 | HBeAb+ | 《100 | yes | ETV+TDF | 0.2 | 5 |
| 29 | male | 67 | HBV | 1 | 20 | yes | yes | √ | √ | √ | √ | √ | √ | √ | √ | √ | RFA | 399 | yes | 18 | HBeAb+ | 《10 | no | ETV | 0.3 | 5 |
| 30 | male | 66 | HBV | 1 | 36 | yes | yes | √ | √ | √ | √ | √ |  |  |  |  | MWA | 425 | no | 19.2 | HBeAb+ | 《100 | no | ETV | 0.3 | 5 |
| 31 | male | 69 | HBV | 1 | 17 | no | yes | √ | √ | √ | √ | √ |  |  |  |  | MWA | 441 | yes | 2.22 | HBeAb+ | 《100 | no | ETV | 0.5 | 5 |
| 32 | male | 64 | HBV | 1 | 17 | no | yes | √ | √ | √ | √ | √ | √ | √ | √ |  | MWA | 266 | yes | 2.16 | HBeAb+ | 《100 | no | ETV | 0.4 | 5 |
| 33 | male | 56 | HBV | 1 | 39 | no | no | √ | √ | √ | √ | √ | √ | √ | √ | √ | MWA | 49 | yes | 818 | HBeAb+ | 《10 | no | ETV | 0.2 | 5 |
| 34 | male | 63 | HBV | 1 | 26 | yes | yes | √ |  | √ | √ |  | √ |  |  |  | MWA | 168 | yes | 1.52 | HBeAb+ | 《100 | no | ETV | 0.1 | 5 |
| 35 | male | 46 | HBV | 1 | 17 | no | yes | √ | √ | √ | √ | √ | √ | √ | √ |  | MWA | 638 | yes | 1.55 | HBeAb+ | 《10 | no | ETV | 0.1 | 5 |
| 36 | male | 64 | HBV | 2 | 40 | yes | yes | √ | √ | √ | √ | √ | √ | √ | √ |  | MWA | 105 | yes | 7.76 | HBeAb+ | 《10 | yes | LAM+ADV | 0.7 | 6 |
| 37 | male | 77 | HBV | 1 | 30 | no | yes | √ |  | √ | √ |  | √ |  |  | √ | MWA | 298 | no | 1.83 | HBeAb+ | 《10 | no | ETV | 0.5 | 5 |
| 38 | male | 66 | HBV | 1 | 30 | yes | yes | √ |  | √ | √ |  | √ |  |  |  | MWA | 72 | yes | 48.1 | HBeAb+ | 28 | no | TAF | 0.8 | 5 |
| 39 | male | 51 | HBV | 1 | 22 | yes | yes | √ | √ | √ | √ | √ | √ |  |  | √ | MWA | 238 | yes | 17694 | HBeAb+ | 《10 | yes | ETV | 0.1 | 5 |
| 40 | male | 58 | HBV | 1 | 14 | yes | yes | √ | √ | √ | √ | √ | √ | √ | √ |  | MWA | 283 | yes | 16.47 | HBeAg+ | 42 | yes | TDF | 0.2 | 5 |
| 41 | female | 66 | HBV | 1 | 15 | yes | yes | √ | √ | √ | √ | √ | √ |  |  |  | MWA | 350 | yes | 18.2 | HBeAb+ | 《100 | no | ETV+TDF | 0.3 | 5 |
| 42 | female | 58 | HBV | 1 | 40 | no | yes | √ | √ | √ | √ | √ | √ |  |  |  | MWA | 227 | no | 21856 | HBeAb+ | 4.75*10*2 | no | ETV | 0.3 | 5 |
| 43 | female | 44 | HBV | 1 | 12 | yes | yes | √ | √ | √ | √ | √ | √ |  |  |  | MWA | 368 | no | 89.6 | HBeAb+ | 《10 | no | TDF | 0.1 | 5 |
| 44 | male | 57 | HBV | 1 | 9 | yes | yes | √ | √ | √ | √ | √ | √ | √ | √ | √ | MWA | 423 | no | 14.5 | HBeAb+ | 《10 | no | TAF | 0.1 | 5 |
| 45 | male | 61 | HBV | 1 | 21 | yes | yes | √ | √ | √ | √ | √ | √ |  |  |  | MWA | 235 | no | 2.33 | HBeAb+ | 《10 | no | ETV | 0.1 | 5 |
| 46 | male | 71 | HBV | 1 | 39 | yes | yes | √ | √ | √ | √ | √ | √ |  |  |  | MWA | 361 | yes | 50.4 | HBeAg+ | 《10 | no | ETV | 0.2 | 5 |
| 47 | male | 65 | HBV | 1 | 30 | no | yes | √ |  | √ | √ |  | √ |  |  |  | MWA | 251 | yes | 1096 | HBeAg+ | 《10 | no | ETV | 0.2 | 5 |
| 48 | male | 49 | HBV | 1 | 21 | no | yes |  | √ | √ | √ | √ |  |  |  |  | MWA | 192 | yes | 2.91 | HBeAb+ | 《10 | no | ETV | 0.4 | 5 |
| 49 | male | 37 | HBV | 1 | 13 | no | yes | √ | √ | √ | √ | √ | √ | √ | √ |  | MWA | 491 | no | 117 | HBeAg+ | 2.53*10*2 | no | ETV | 0.1 | 5 |
| 50 | male | 69 | HBV | 1 | 20 | no | yes | √ |  | √ | √ |  | √ |  |  | √ | MWA | 250 | no | 1.69 | HBeAb+ | 《100 | no | ETV | 0.2 | 5 |
| 51 | male | 42 | HBV | 1 | 20 | no | yes | √ |  | √ | √ |  | √ |  |  |  | MWA | 297 | no | 255 | HBeAg+ | 68 | no | TAF | 0.3 | 5 |
| 52 | male | 61 | HBV | 1 | 25 | no | yes | √ |  |  | √ |  |  |  |  |  | MWA | 214 | no | 2.28 | HBeAb+ | 《100 | no | ETV | 0.3 | 5 |
| 53 | female | 62 | HBV | 1 | 22 | no | yes | √ | √ | √ | √ | √ | √ | √ | √ | √ | MWA | 384 | yes | 4.32 | HBeAb+ | 《100 | no | ETV | 0.5 | 5 |
| 54 | male | 70 | HBV | 2 | 40 | yes | yes | √ |  | √ | √ |  | √ |  |  |  | MWA | 335 | yes | 22.8 | HBeAg+ | 《100 | no | TDF | 0.3 | 5 |
| 55 | male | 83 | HBV | 1 | 16 | yes | yes | √ | √ | √ | √ | √ |  | √ | √ |  | MWA | 374 | no | 3.13 | HBeAb+ | 《10 | no | ETV | 0.2 | 5 |
| 56 | male | 53 | HBV | 1 | 9 | yes | yes | √ | √ | √ | √ | √ | √ |  |  |  | MWA | 118 | yes | 33.2 | HBeAg+ | 8015 | yes | TDF | 0.8 | 5 |

We recruited 56 patients with HBV-related HCC whose liver function was Child A and BCLN grade A stage. All subjects without ongoing co-infections or other diseases . Ratio of males to females: 44/12, average age: 61 years old, Child-Pugh :5/6(51/5), No. of nodules :1/2（52/4），Cumulative diameter: 22.5mm(9-40), A single course of treatment for the tumor ablation, the melting edge of the edge is 5-10 millimeters is the whole goal. At the end of the operation, the ablation area was enhanced by enhanced CT. According to the operator's judgment, the CT enhancement control scan is immediately performed, if the edge <5mm, can expand the edge, to ensure complete necrosis(56). RFS (recurrence-free survival) was calculated from the first ablation and follow-up was calculated from the beginning of ablation to the last follow-up. After a median follow-up of 290 days(49-664), 26 had tumor recurrence , the median time for recurrences was 384 days. Available data Phenotypic characterization D0(55),D7(56),M1(14). Intracellular cytokine staining D0(32),D7(32),M1(14). Cytotoxicity (K562) D0(54),D7(48),M1(10).
