# Supplementary material for: Dynamic changes of phenotype and function of natural killer cells in peripheral blood before and after thermal ablation of hepatitis B associated hepatocellular carcinoma and their correlation with tumor recurrence
Source: BMC Cancer. 2023 May 30;23:486. doi: 10.1186/s12885-023-10823-4 (PMC10228897; doi:10.1186/s12885-023-10823-4)
Supplement: Supplementary file 2 — Additional file 2. [file 12885_2023_10823_MOESM2_ESM.docx]

date of ADCC（LDH）

| Patient no. | Gender | Age (y) | Cytotoxicity (K562) D0 | Cytotoxicity (K562) D7 | Cytotoxicity (K562) M1 |
| --- | --- | --- | --- | --- | --- |
| 1 | male | 57 | 0.626109 | 0.826362 | 0.59856 |
| 2 | male | 55 | 0.98939 | 1.550398 |  |
| 3 | male | 74 |  | 0.295276 |  |
| 4 | female | 66 | 0.415282 | 0.826087 | 0.38412 |
| 5 | female | 47 | 0.296154 |  |  |
| 6 | male | 71 | 0.63314 | 0.834963 |  |
| 7 | male | 63 | 0.473388 | 0.562238 |  |
| 8 | male | 58 | 0.5 |  |  |
| 9 | female | 70 | 0.225602 | 0.585551 |  |
| 10 | male | 58 | 0.638783 | 0.664132 |  |
| 11 | female | 66 | 0.106101 | 0.962865 |  |
| 12 | male | 62 | 0.4912 | 0.672956 |  |
| 13 | male | 68 | 0.579576 | 0.892573 | 0.47834 |
| 14 | male | 47 | 0.393195 | 0.62949 |  |
| 15 | male | 56 | 0.396875 | 0.504587 |  |
| 16 | female | 68 | 0.247706 | 0.7875 |  |
| 17 | male | 61 | 0.72619 | 0.813462 |  |
| 18 | male | 54 | 1.119093 | 1.821803 |  |
| 19 | female | 46 | 0.861635 | 1.226415 |  |
| 20 | male | 66 | 0.423741548 | 0.311538462 |  |
| 21 | male | 63 | 0.710875 | 0.793103 |  |
| 22 | male | 55 | 1.201258 | 2.138365 |  |
| 23 | male | 59 | 0.224771 | 0.426606 |  |
| 24 | male | 60 | 0.457467 | 0.6864 |  |
| 25 | female | 52 | 0.354713 | 0.597184 |  |
| 26 | female | 61 | 0.48009 | 0.672727 |  |
| 27 | male | 67 | 0.593573 | 0.761006 |  |
| 28 | male | 66 | 0.106781 |  |  |
| 29 | male | 67 | 0.412587413 | 0.573076923 | 0.310293013 |
| 30 | male | 66 | 0.307087 |  |  |
| 31 | male | 69 | 1.404762 |  |  |
| 32 | male | 64 | 1.452381 | 4.631579 |  |
| 33 | male | 56 | 0.880952 | 1.719231 | 0.515385 |
| 34 | male | 63 | 0.430925 | 1.046895 |  |
| 35 | male | 46 | -1.59184 | 1.461538 |  |
| 36 | male | 64 | 0.368119 | 0.753125 |  |
| 37 | male | 77 | 0.579576 | 0.892573 | 0.54562 |
| 38 | male | 66 | 0.5 | 0.639375 |  |
| 39 | male | 51 | 0.190945 | 0.319231 | 0.15412 |
| 40 | male | 58 | 0.646825 | 1.988764 |  |
| 41 | female | 66 | 0.919291 | 0.953846 |  |
| 42 | female | 55 | 0.271803 | 0.380524 |  |
| 43 | female | 44 | 0.477085 | 0.506294 |  |
| 44 | male | 57 | 0.66717 | 0.409615 | 0.177165 |
| 45 | male | 61 | 0.325772 | 0.420415 |  |
| 46 | male | 71 | 0.517857 | 0.506294 |  |
| 47 | male | 65 | 0.68244 | 0.860774 |  |
| 48 | male | 49 | 0.17629 |  |  |
| 49 | male | 37 | 1.139721 | 1.431935 |  |
| 50 | male | 69 | 1.121593 | 1.574423 | 0.98732 |
| 51 | male | 42 | 0.510397 | 0.620545 |  |
| 52 | male | 61 |  |  |  |
| 53 | female | 62 | 0.186538 | 0.27972 | 0.503846 |
| 54 | male | 70 | 0.896552 | 3.723618 |  |
| 55 | male | 83 | 0.315552 |  |  |
| 56 | male | 53 | 0.225602 | 0.250951 |  |

Fifty-six patients clinically and pathologically confirmed with hepatitis B associated hepatocellular carcinoma (HCC) were selected for thermal ablation. Peripheral blood was collected from patients isolated on the D0, D7and month M1. NK cell subsets, receptors and killing function were detected by flow cytometry and the LDH. NK cell activity was detected by LDH release assay. Resuscitate the PBMC and wash it once, lay a six-well plate in a 37° incubator overnight, collect cells, filter 70 mesh, centrifuge, discard supernatant, count cells, adjust cell concentration 100ul containing 5*105, K562 cell concentration 100ul containing 5*103(efficiency target ratio 100: 1) ,96-well plates were mixed in a 37° incubator overnight. Each specimen was set with 3 multiple Wells, and the natural release control group and the maximum release control group of target cells were set at the same time. 10ul water was added to each natural release well, and 10ul lysate was added to each maximum release well, and the 37° incubator lasted 45 minutes. Transfer 50ul supernatant per well to a new 96-well plate, add 50ul reaction Solution (600ul Assay Buffer+ 11.4 mL Substrate Stock Solution) to each well, room temperature for 30min, add 50ul stop Solution 490nm to test OD. The percentage of cytotoxicity was calculated using the following formula: cytotoxicity (%) = (test sample - low control) / (high control - low control) %.
