# Supplementary material for: Dynamic changes of phenotype and function of natural killer cells in peripheral blood before and after thermal ablation of hepatitis B associated hepatocellular carcinoma and their correlation with tumor recurrence
Source: BMC Cancer. 2023 May 30;23:486. doi: 10.1186/s12885-023-10823-4 (PMC10228897; doi:10.1186/s12885-023-10823-4)
Supplement: Supplementary file 3 — Additional file 3. [file 12885_2023_10823_MOESM3_ESM.docx]

Flow cytometry data of patients

| no. | no. | Lymphocytes/Single Cells \| Freq. of Parent（%） | Lymphocytes/Single Cells/CD3- \| Freq. of Parent（%） | Lymphocytes/Single Cells/CD3-/CD3-CD56+ \| Freq. of Parent（%） | Lymphocytes/Single Cells/CD3-/CD3-CD56+/CD56+CD16+ \| Freq. of Parent（%） | Lymphocytes/Single Cells/CD3-/CD3-CD56+/CD56+CD158a+ \| Freq. of Parent（%） | Lymphocytes/Single Cells/CD3-/CD3-CD56+/CD56+CD158b+ \| Freq. of Parent（%） | Lymphocytes/Single Cells/CD3-/CD3-CD56+/CD56+CD159a+ \| Freq. of Parent（%） | Lymphocytes/Single Cells/CD3-/CD3-CD56+/CD56+NKG2D+ \| Freq. of Parent（%） | Lymphocytes/Single Cells/CD3-/CD3-CD56+/CD56+NKp30+ \| Freq. of Parent（%） | Lymphocytes/Single Cells/CD3-/CD3-CD56+/CD56+NKp46+ \| Freq. of Parent（%） | Lymphocytes/CD3- \| Freq. of Parent（%） | Lymphocytes/CD3-/CD3-CD56+ \| Freq. of Parent（%） | Lymphocytes/CD3-/CD3-CD56+/CD56+Grazm B+ \| Freq. of Parent（%） | Lymphocytes/CD3-/CD3-CD56+/CD56+IFN-gamma+ \| Freq. of Parent（%） | Lymphocytes/CD3-/CD3-CD56+/CD56+perforin+ \| Freq. of Parent（%） |
| --- | --- | --- | --- | --- | --- | --- | --- | --- | --- | --- | --- | --- | --- | --- | --- | --- |
| 1 | D0 | 75 | 67.2 | 25.8 | 1.05 | 0.09 | 0.56 | 0.2 | 0.022 | 0.25 | 0.47 |  |  |  |  |  |
| 1 | D7 | 69.5 | 55.2 | 34 | 3.3 | 0.54 | 2.48 | 1.01 | 0.68 | 1.08 | 1.8 |  |  |  |  |  |
| 2 | D0 | 59.9 | 52.9 | 31.1 | 10.3 | 0.2 | 2.32 | 0.6 | 0.2 | 1.71 | 0.65 |  |  |  |  |  |
| 2 | D7 | 44 | 84.4 | 38.7 | 25.3 | 1.03 | 6.49 | 11.9 | 8.35 | 4.07 | 2.25 |  |  |  |  |  |
| 3 | D0 | 0.45 | 0 | 72.6 | 15.1 | 75 | 37.5 | 0 | 0 | 0 | 12.5 | 2.58 | 49.6 | 31.2 | 16.7 | 93.8 |
| 3 | D7 | 0.28 | 14.8 | 61.2 | 24.7 | 61.9 | 23.8 | 28.6 | 0 | 0 | 14.3 | 3.55 | 36.6 | 44.1 | 17.6 | 94.1 |
| 4 | D0 | 30.1 | 99.1 | 52.6 | 6.52 | 69.7 | 0.48 | 17.6 | 4.43 | 4.55 | 13.6 | 36 | 47.3 | 92.6 | 54.6 | 2.11 |
| 4 | D7 | 1.01 | 94.4 | 55.2 | 5.09 | 65 | 0 | 5 | 0 | 0 | 15 | 5.44 | 53.5 | 89.7 | 61.2 | 6.9 |
| 4 | M1 | 13.4 | 93.6 | 22 | 1.2 | 70.9 | 0.61 | 24.2 | 17.9 | 15.2 | 6.36 | 28.3 | 14.9 | 97.1 | 86.7 | 7.14 |
| 5 | D0 | 0.13 | 0 | 0 | 0 | 0 | 0 | 0 | 0 | 0 | 0 | 1.91 | 55 | 7.69 | 5.13 | 97.4 |
| 5 | D7 | 17.1 | 11.8 | 82.1 | 5.09 | 67.1 | 0.44 | 14.2 | 92.2 | 22 | 4.58 | 30.3 | 20.3 | 82.4 | 14.3 | 91 |
| 6 | D0 | 54.1 | 63.6 | 39.6 | 6.97 | 1.06 | 3.98 | 3.42 | 2.04 | 3.7 | 1.51 |  |  |  |  |  |
| 6 | D7 | 54.3 | 92.4 | 35 | 4.95 | 0.32 | 1.94 | 1.43 | 0.78 | 2.34 | 1.36 |  |  |  |  |  |
| 7 | D0 | 29.2 | 1.97 | 39.9 | 28.3 | 47.4 | 0.026 | 33.1 | 38 | 14.4 | 6.26 | 53.7 | 93.1 | 1.16 | 52.8 | 83.3 |
| 7 | D7 | 18.7 | 33.2 | 85.7 | 20.6 | 49.8 | 0 | 32.5 | 68.7 | 54.2 | 9.34 | 42.9 | 10.6 | 97.9 | 25.7 | 58.7 |
| 8 | D0 | 71.2 | 27.5 | 1.79 | 56.4 | 1.01 | 37.9 | 58.7 | 28.9 | 2.35 | 9.06 | 24.8 | 38.2 | 48.8 | 3.24 | 83.1 |
| 8 | D7 | 8.22 | 1.28 | 29.7 | 6.68 | 46.5 | 35.3 | 8.21 | 2.43 | 0.3 | 12.8 | 20.8 | 10.3 | 90.6 | 43.5 | 53.9 |
| 9 | D0 | 81.7 | 33.8 | 5.82 | 86.4 | 1.94 | 25.3 | 15.7 | 4.94 | 1.54 | 11.4 | 37.9 | 82 | 57.7 | 2.12 | 90.1 |
| 9 | D7 | 76.3 | 44.3 | 6.68 | 80.8 | 1.43 | 26 | 20.7 | 7.4 | 0.8 | 18.4 | 45.4 | 81.5 | 50.6 | 2.5 | 93.6 |
| 10 | D0 | 73.3 | 58.1 | 36.6 | 6.98 | 0.58 | 2.82 | 1.62 | 0.58 | 1.91 | 1.34 |  |  |  |  |  |
| 10 | D7 | 70.1 | 60.9 | 37.9 | 8.12 | 0.99 | 4.65 | 3.72 | 2.04 | 2.52 | 2.03 |  |  |  |  |  |
| 11 | D0 | 63.3 | 43.9 | 34.9 | 7.21 | 0.25 | 1.21 | 0.97 | 0.21 | 0.6 | 1.32 |  |  |  |  |  |
| 11 | D7 | 81.8 | 31.7 | 39 | 10.6 | 0.69 | 3.29 | 2.06 | 0.33 | 1.34 | 2.03 |  |  |  |  |  |
| 12 | D0 | 81.8 | 86.6 | 31.2 | 6.64 | 0.27 | 0.8 | 0.53 | 0.12 | 0.63 | 0.85 |  |  |  |  |  |
| 12 | D7 | 70.9 | 48.4 | 37.7 | 1.06 | 0.81 | 3.14 | 2.49 | 1.11 | 4.01 | 1.87 |  |  |  |  |  |
| 13 | D0 | 74.8 | 70.8 | 35.8 | 4.94 | 0.74 | 3.19 | 1.91 | 0.73 | 1.34 | 1.47 |  |  |  |  |  |
| 13 | D7 | 67.5 | 61.5 | 36.4 | 11.1 | 0.6 | 2.72 | 1.79 | 0.78 | 1.21 | 1.44 |  |  |  |  |  |
| 14 | D0 | 71.4 | 72.1 | 36.8 | 1.61 | 0.75 | 3.46 | 3.56 | 2.16 | 2.04 | 1.55 |  |  |  |  |  |
| 14 | D7 | 86.5 | 46.8 | 32.7 | 1.06 | 0.25 | 0.74 | 0.39 | 0.21 | 0.99 | 0.92 |  |  |  |  |  |
| 15 | D0 | 67.9 | 57.1 | 5 | 50.4 | 0 | 12.4 | 69.9 | 5.01 | 0.15 | 39.7 | 51.1 | 64.7 | 44.2 | 2.59 | 82.6 |
| 15 | D7 | 77.6 | 25.9 | 8.41 | 59.1 | 0.22 | 12.8 | 71.9 | 5.24 | 0.44 | 39.7 | 22.1 | 52.4 | 35 | 2.45 | 72.1 |
| 16 | D0 | 52.2 | 78.5 | 30.4 | 0.81 | 0.19 | 1 | 0.4 | 0.24 | 1 | 0.52 |  |  |  |  |  |
| 16 | D7 | 87.4 | 42.1 | 43.7 | 9.32 | 1.37 | 7.09 | 9.15 | 5.15 | 3.09 | 5.37 |  |  |  |  |  |
| 17 | D0 | 20.3 | 1.2 | 10.8 | 16.6 | 53.6 | 30.2 | 19.4 | 1.89 | 0 | 26.3 | 45.4 | 17.4 | 96.9 | 61.6 | 37.7 |
| 17 | D7 | 10.4 | 8.49 | 28.9 | 20.2 | 66.9 | 22 | 16.1 | 0.76 | 0 | 14.6 | 57.9 | 36.2 | 96.4 | 59.1 | 39.2 |
| 17 | M1 | 5.9 | 7.34 | 20.3 | 46.1 | 58.4 | 25.7 | 11.5 | 0.29 | 0 | 4 | 28.2 | 19.1 | 95.6 | 52.2 | 31.4 |
| 18 | D0 | 17.2 | 52.3 | 7.69 | 64.8 | 0 | 22.5 | 45.1 | 9.86 | 0 | 14.1 | 3.54 | 40.5 | 37.9 | 3.76 | 63.6 |
| 18 | D7 | 73.7 | 30.4 | 14.6 | 84.1 | 1.77 | 35.1 | 26 | 5.73 | 0.8 | 18.7 | 37.3 | 83.2 | 61.6 | 2.08 | 95.8 |
| 19 | D0 | 68 | 51.6 | 31.2 | 5.25 | 0.25 | 1.08 | 0.29 | 0.12 | 1.21 | 1.04 |  |  |  |  |  |
| 19 | D7 | 58.9 | 65.9 | 40.2 | 1.95 | 1.44 | 7.16 | 11.6 | 8.6 | 5.92 | 3.9 |  |  |  |  |  |
| 20 | D0 | 76.7 | 43.1 | 2.64 | 69.8 | 0.36 | 42.2 | 36.4 | 21.8 | 1.45 | 19.6 | 38.1 | 52.4 | 55.3 | 2.4 | 85.5 |
| 20 | D7 | 12.5 | 5.66 | 93.1 | 2.96 | 51.9 | 0.18 | 11.6 | 64.9 | 44.5 | 3.33 | 22.7 | 18.8 | 2.3 | 47.3 | 55 |
| 20 | M1 | 7.27 | 18.9 | 92.2 | 11 | 30.8 | 0.045 | 18.8 | 81.1 | 65.4 | 7.27 | 23.7 | 12 | 96.4 | 66.6 | 18.1 |
| 21 | D0 | 39.5 | 52.8 | 28.4 | 1.9 | 0.12 | 0.74 | 0.31 | 0.31 | 4.05 | 1.29 |  |  |  |  |  |
| 21 | D7 | 40.8 | 47.6 | 39.4 | 4.29 | 0.3 | 3.95 | 9.33 | 6.07 | 8.84 | 2.02 |  |  |  |  |  |
| 22 | D0 | 39.2 | 78.2 | 32.6 | 1.36 | 0.62 | 2.39 | 1.95 | 1.29 | 6.06 | 1.18 |  |  |  |  |  |
| 22 | D7 | 48.7 | 77.1 | 32.9 | 1.98 | 0.51 | 3.05 | 1.43 | 0.59 | 5.51 | 1.45 |  |  |  |  |  |
| 23 | D0 | 64 | 72.1 | 28.8 | 3.77 | 0.19 | 1.29 | 0.19 | 0.12 | 0.92 | 0.6 |  |  |  |  |  |
| 23 | D7 | 73.5 | 52.5 | 34.7 | 10.8 | 0.4 | 3.14 | 0.45 | 0.23 | 2.12 | 1.05 |  |  |  |  |  |
| 24 | D0 | 57.1 | 41 | 26 | 2.18 | 0.16 | 0.6 | 0.16 | 0 | 0.76 | 0.76 |  |  |  |  |  |
| 24 | D7 | 82 | 34.7 | 37 | 2.33 | 0.51 | 1.75 | 1.24 | 0.8 | 1.46 | 1.31 |  |  |  |  |  |
| 25 | D0 | 57.9 | 73.7 | 38.3 | 7.29 | 0.49 | 4.49 | 3.86 | 2.63 | 2.81 | 2.42 |  |  |  |  |  |
| 25 | D7 | 47.4 | 95.1 | 38.9 | 0.81 | 0.25 | 2.96 | 4.51 | 4 | 2.69 | 0.62 |  |  |  |  |  |
| 26 | D0 | 15.5 | 30.9 | 89 | 18.3 | 47.4 | 0.24 | 29 | 69.1 | 47.3 | 15.5 | 39.2 | 13.5 | 99 | 41.6 | 16 |
| 26 | D7 | 13.9 | 25.5 | 93.7 | 18.9 | 45.7 | 0.36 | 37.3 | 68.2 | 46.9 | 17.5 | 36.6 | 13.9 | 99.9 | 44.8 | 14.4 |
| 27 | D0 | 72 | 83.6 | 38 | 3.09 | 0.69 | 3.46 | 3.92 | 2.23 | 1.4 | 1.71 |  |  |  |  |  |
| 27 | D7 | 83.5 | 79.6 | 37.9 | 3.37 | 0.78 | 2.6 | 2.65 | 0.97 | 1.66 | 1.28 |  |  |  |  |  |
| 28 | D0 | 76.1 | 26.6 | 1.66 | 19.4 | 0 | 28.1 | 46.2 | 17 | 0.91 | 21.3 | 18.8 | 10.9 | 38.4 | 3.77 | 53.6 |
| 28 | D7 | 21.8 | 3.79 | 9.08 | 3.95 | 95 | 24.1 | 5.67 | 3.55 | 0 | 5.67 | 49.1 | 23.4 | 98 | 65 | 17.5 |
| 28 | M1 | 65.5 | 32.4 | 5.47 | 78.6 | 2.18 | 37.2 | 17.6 | 6.3 | 2.06 | 25.1 | 28.1 | 60.5 | 42.7 | 3.02 | 75.2 |
| 29 | D0 | 2.73 | 22.2 | 68.1 | 28.7 | 93.8 | 1.17 | 7.03 | 48.1 | 2.69 | 3.38 | 11.8 | 27.8 | 96.8 | 71.3 | 27.1 |
| 29 | D7 | 4.11 | 34.2 | 89.4 | 26.9 | 56.7 | 0.84 | 16.2 | 39.6 | 24.3 | 6.73 | 20.9 | 23.6 | 98.9 | 87.2 | 7.38 |
| 29 | M1 | 33 | 39.5 | 88.7 | 37.1 | 29.7 | 0.26 | 29.9 | 71.2 | 61.4 | 5.36 | 63.5 | 65.9 | 98.6 | 63.2 | 1.48 |
| 30 | D0 | 0.063 | 0 | 76.9 | 31.4 | 63.6 | 0 | 9.09 | 86.4 | 27.3 | 9.09 | 1.35 | 36 | 66 | 12.8 | 8.51 |
| 30 | D7 | 0.071 | 0 | 100 | 0 | 0 | 0 | 0 | 0 | 0 | 0 | 1.94 | 43.8 | 24 | 28 | 88 |
| 31 | D0 | 9.09 | 3.92 | 15.9 | 6.99 | 94.2 | 0.83 | 0.83 | 67.2 | 3.33 | 0.56 | 39.9 | 21.5 | 97.3 | 63.1 | 36.4 |
| 31 | D7 | 0.25 | 0 | 83.1 | 13.7 | 51.5 | 0 | 39.4 | 63.6 | 45.5 | 6.06 | 3.6 | 42.4 | 14.3 | 13.2 | 92.3 |
| 32 | D0 | 10.3 | 94.7 | 28.6 | 0.66 | 23.1 | 0 | 7.69 | 0 | 0 | 0 | 28.4 | 8.99 | 86.2 | 50.8 | 5.14 |
| 32 | D7 | 2.27 | 87.1 | 57.4 | 3.37 | 66.7 | 0 | 33.3 | 0 | 0 | 0 | 8.67 | 52 | 75.2 | 34.7 | 3.96 |
| 32 | M1 | 25.1 | 99.3 | 27.7 | 4.17 | 79.3 | 6.14 | 32.2 | 8.95 | 12 | 19.7 | 25.5 | 14.6 | 98.9 | 81.8 | 9.42 |
| 33 | D0 | 7.21 | 1.77 | 11.9 | 3.18 | 61.9 | 23.8 | 11.9 | 4.76 | 0 | 28.6 | 17.4 | 17.3 | 91.4 | 23 | 79.9 |
| 33 | D7 | 17.5 | 30 | 90.9 | 14.4 | 28.2 | 0.05 | 38.7 | 85 | 68.7 | 11.3 | 51.2 | 10.7 | 93.1 | 47.7 | 13.6 |
| 33 | M1 | 2.05 | 1.79 | 13.3 | 12.5 | 68.8 | 50 | 10.9 | 9.38 | 0 | 9.38 | 9.95 | 11 | 97.4 | 49.9 | 27.4 |
| 34 | D0 | 39.8 | 94.4 | 29.6 | 1.15 | 0.1 | 1.25 | 0.5 | 0.39 | 0.89 | 0.91 |  |  |  |  |  |
| 34 | D7 | 32.6 | 95.3 | 37.5 | 6.8 | 0.62 | 4.54 | 6.52 | 3.71 | 3.64 | 2.39 |  |  |  |  |  |
| 35 | D0 | 0.39 | 62.7 | 34.4 | 0 | 0 | 0 | 0 | 0 | 0 | 0 | 4.06 | 42.9 | 50 | 33.3 | 0 |
| 35 | D7 | 16.9 | 98 | 36.9 | 2.39 | 55.1 | 0 | 22.7 | 1.83 | 3.43 | 23.1 | 33.5 | 91 | 0.38 | 56 | 1.67 |
| 35 | M1 | 18.9 | 98.1 | 40.7 | 7.69 | 87.2 | 2.54 | 42.4 | 22.1 | 13.3 | 18.7 | 28.7 | 30.1 | 96.8 | 84 | 7.82 |
| 36 | D0 | 78.2 | 65.2 | 1.51 | 40.4 | 0.35 | 22.3 | 65.2 | 17 | 6.03 | 24.8 | 43.2 | 25.8 | 64.8 | 3.42 | 77.4 |
| 36 | D7 | 68.4 | 61.3 | 4.13 | 39.9 | 0.25 | 17.2 | 62.9 | 8.78 | 3.4 | 28.5 | 44.8 | 42.1 | 56.3 | 3.51 | 71.1 |
| 36 | M1 | 81 | 18.7 | 2.21 | 53.2 | 0 | 23.4 | 64.7 | 14 | 2.55 | 41.7 | 20.2 | 50.1 | 50.1 | 2.37 | 81.4 |
| 37 | D0 | 76.6 | 63.8 | 37.1 | 3.02 | 0.61 | 2.83 | 1.63 | 0.93 | 1.06 | 1.8 |  |  |  |  |  |
| 37 | D7 | 80.3 | 63.7 | 37.4 | 2.66 | 0.71 | 3.64 | 2.31 | 1.07 | 1.28 | 1.87 |  |  |  |  |  |
| 38 | D0 | 59.6 | 78.8 | 31.3 | 7.78 | 0.21 | 1.19 | 0.82 | 0.47 | 1.14 | 0.95 |  |  |  |  |  |
| 38 | D7 | 71 | 83.4 | 33.7 | 2.28 | 0.3 | 1.56 | 1.16 | 0.38 | 1.13 | 1.57 |  |  |  |  |  |
| 39 | D0 | 22.6 | 0.54 | 11.7 | 21 | 60.2 | 50.8 | 21.1 | 1.75 | 0 | 41 | 60.3 | 12.9 | 92.4 | 50.8 | 52.5 |
| 39 | D7 | 1 | 0 | 42.8 | 19 | 26.1 | 56.5 | 17.4 | 0 | 0 | 17.4 | 5.43 | 28.3 | 25 | 16.7 | 97.2 |
| 40 | D0 | 1.53 | 98.3 | 16.8 | 5.26 | 0 | 0 | 0 | 100 | 100 | 0 | 8.92 | 20.8 | 78.6 | 71.4 | 17.9 |
| 40 | D7 | 16.6 | 98 | 35.5 | 1.18 | 55.9 | 0 | 25.5 | 11.3 | 13.7 | 27 | 25.7 | 28.4 | 94.9 | 44.7 | 4.55 |
| 40 | M1 | 39.7 | 98.3 | 26.4 | 4.97 | 75.2 | 0.065 | 30 | 10.1 | 15.2 | 28.9 | 56.7 | 22.9 | 88.4 | 40.9 | 4.1 |
| 41 | D0 | 2.2 | 0 | 91.6 | 15.2 | 31.9 | 0 | 44.7 | 90.1 | 61.7 | 10.6 | 26.8 | 22.8 | 54.1 | 8.16 | 82.7 |
| 41 | D7 | 24.9 | 7.14 | 92.6 | 10.1 | 31.9 | 0.14 | 38.2 | 81 | 65.6 | 18 | 42.1 | 20.3 | 93.7 | 12.9 | 73.6 |
| 42 | D0 | 44 | 7.35 | 1.17 | 100 | 0 | 100 | 100 | 100 | 0 | 0 | 0.63 | 29.6 | 37.9 | 15.2 | 48.5 |
| 42 | D7 | 46.9 | 2.68 | 2.06 | 22.2 | 0 | 55.6 | 66.7 | 55.6 | 0 | 0 | 0.85 | 21.3 | 43.1 | 5.56 | 19.4 |
| 43 | D0 | 13.4 | 16.4 | 89.8 | 8.59 | 50.2 | 0.13 | 18.6 | 77 | 39.3 | 4.63 | 40.6 | 14.6 | 81.3 | 26 | 31 |
| 43 | D7 | 10.1 | 6.15 | 94.8 | 4.11 | 64.4 | 0.26 | 19.1 | 60.9 | 30.4 | 5.96 | 32.3 | 25.2 | 8.34 | 16.5 | 63 |
| 44 | D0 | 14.1 | 5.76 | 21.6 | 17 | 55.7 | 40.5 | 12.9 | 2.86 | 0 | 21 | 42.7 | 9.63 | 90.1 | 60.1 | 44.2 |
| 44 | D7 | 17.4 | 0 | 96.2 | 4.18 | 76.2 | 0.26 | 7.46 | 64 | 17.5 | 23.8 | 37.1 | 28 | 90.4 | 39.1 | 63.3 |
| 44 | M1 | 10.2 | 19.4 | 79.2 | 15.9 | 43 | 0.053 | 17.7 | 74.9 | 45.8 | 8.94 | 28.4 | 13.5 | 87.7 | 75.3 | 22.3 |
| 45 | D0 | 65.7 | 19.9 | 2.72 | 59 | 0.21 | 35.5 | 36.5 | 14.6 | 0.41 | 25.4 | 19.1 | 35.4 | 49.7 | 2.79 | 80.7 |
| 45 | D7 | 57.5 | 23.8 | 4.59 | 51.9 | 0.59 | 45.3 | 37.6 | 23.1 | 1.04 | 16 | 23.3 | 42 | 53 | 3.88 | 79.3 |
| 46 | D0 | 11 | 1.13 | 13.5 | 11.5 | 43.9 | 23.6 | 2.03 | 2.03 | 0 | 1.35 | 38 | 19.1 | 95.2 | 71.2 | 26.1 |
| 46 | D7 | 20.1 | 50 | 92.4 | 5.59 | 56.2 | 0.24 | 19.6 | 60 | 40 | 3.39 | 44.4 | 23.4 | 10.8 | 44 | 36.6 |
| 47 | D0 | 51.3 | 85.8 | 28.7 | 3.55 | 0.16 | 1.76 | 0.35 | 0.073 | 1.58 | 0.4 |  |  |  |  |  |
| 47 | D7 | 49.9 | 69.7 | 36.7 | 4.74 | 1.05 | 5.58 | 4.53 | 2.18 | 4.11 | 2.85 |  |  |  |  |  |
| 48 | D0 |  |  |  |  |  |  |  |  |  |  | 2.42 | 47.8 | 15.6 | 3.33 | 97.8 |
| 48 | D7 | 8.32 | 13 | 93.2 | 7.11 | 59.5 | 0.33 | 10.7 | 66.1 | 35.6 | 11.5 | 24.2 | 11.3 | 97.7 | 24.8 | 44.9 |
| 49 | D0 | 47.8 | 95.7 | 48.1 | 33 | 3.56 | 0.046 | 54.6 | 88 | 91.2 | 5.77 | 58.2 | 32.9 | 90.7 | 34.8 | 1.13 |
| 49 | D7 | 20 | 91.2 | 69 | 39.3 | 51.9 | 0.054 | 58 | 92.6 | 97.4 | 2.16 | 32.6 | 64.9 | 84 | 29.8 | 3.79 |
| 49 | M1 | 21.5 | 94 | 31.6 | 2.84 | 74.9 | 1.63 | 29.2 | 9.56 | 19.8 | 11.8 | 23.5 | 28.7 | 92.6 | 79.2 | 9.03 |
| 50 | D0 | 70.9 | 36.9 | 36.8 | 2.05 | 0.87 | 2.75 | 1.44 | 0.87 | 1.96 | 1.83 |  |  |  |  |  |
| 50 | D7 | 43.1 | 75.6 | 31.1 | 0.25 | 0.3 | 1.09 | 0.37 | 0.17 | 0.87 | 1.16 |  |  |  |  |  |
| 51 | D0 | 59.4 | 80.1 | 32.1 | 1.78 | 0.22 | 1.47 | 0.31 | 0.16 | 0.59 | 0.87 |  |  |  |  |  |
| 51 | D7 | 88.1 | 49.4 | 30.3 | 3.82 | 0.27 | 2.03 | 0.47 | 0.12 | 0.7 | 1.13 |  |  |  |  |  |
| 52 | D0 | 87.8 | 81.9 | 49.5 | 19.4 | 10.9 | 64.8 | 61.9 | 72.9 | 50.2 | 10.1 |  |  |  |  |  |
| 52 | D7 | 84.6 | 84.7 | 42.7 | 1.84 | 1.28 | 9.57 | 17.7 | 17.1 | 7.22 | 2.32 |  |  |  |  |  |
| 53 | D0 | 11.5 | 8.41 | 35.8 | 27.2 | 68.5 | 27.1 | 26.6 | 0.49 | 0 | 7.35 | 43.3 | 24 | 83.5 | 41.9 | 73.9 |
| 53 | D7 | 16.9 | 0 | 95.5 | 8.36 | 58.9 | 0 | 25.1 | 72.8 | 33.1 | 10.1 | 44 | 24.2 | 93.6 | 15.4 | 69.1 |
| 53 | M1 | 13 | 10.4 | 92.7 | 7.13 | 25.7 | 0.12 | 44.9 | 93.2 | 84.8 | 3.37 | 30.1 | 17 | 93.1 | 29 | 44.3 |
| 54 | D0 | 44.4 | 91.9 | 24.2 | 0.61 | 0.083 | 0.83 | 0.14 | 0.11 | 0.66 | 0.61 |  |  |  |  |  |
| 54 | D7 | 49.3 | 80.1 | 33.7 | 9.85 | 0.17 | 21.1 | 7.69 | 11.8 | 11 | 22.2 |  |  |  |  |  |
| 55 | D0 | 3.57 | 1.73 | 29.7 | 14.5 | 98.6 | 23.3 | 6.85 | 1.37 | 0 | 34.2 | 8.24 | 35.5 | 93.9 | 43.8 | 75.8 |
| 55 | D7 | 18.8 | 5.97 | 59.9 | 60.8 | 57.5 | 13 | 8.94 | 0 | 0 | 0.19 | 51.4 | 65.6 | 94.9 | 79.8 | 5.22 |
| 55 | M1 | 19.1 | 2.14 | 32.8 | 43.7 | 55.7 | 26 | 9.86 | 9.17 | 0.52 | 0.35 | 54.4 | 6.2 | 90.4 | 41.8 | 68.7 |
| 56 | D0 | 78.4 | 44.1 | 2.94 | 53.8 | 5.49 | 28.6 | 13.2 | 0 | 6.59 | 20.9 | 25.7 | 36.4 | 75.2 | 1.38 | 74.7 |
| 56 | D7 | 72.8 | 45 | 8.7 | 47.4 | 2.29 | 25.3 | 27.9 | 4 | 0.91 | 26.1 | 35.6 | 62.6 | 68.1 | 3.9 | 77.3 |

Fifty-six patients clinically and pathologically confirmed with hepatitis B associated hepatocellular carcinoma (HCC) were selected for thermal ablation. Peripheral blood was collected from patients isolated on the D0, D7and month M1. NK cell subsets, receptors and killing function were detected by flow cytometry. Peripheral blood mononuclear cells (PBMCs) were thawed in a water bath at 37°C for one minute, washed and resuspended in Roswell Park Memorial Institute (RPMI) medium containing 10% v/v fetal calf serum. Antibodies were incubated at 4°C for surface staining, and PBMCs were stained with the following fluorophore-conjugated human monoclonal antibodies at room temperature for 20 min: anti-CD3-APC-H7,anti-CD56-BB515,anti-CD16-PE(Figure1A),anti-NKp46-PE-cy7, anti-NKp30-BV421, anti-CD158a-APC,anti-CD158b-BV785,anti-NKG2D-BV605,anti-CD159a-BB700（Figure2A);For intracellular staining, the cells were permeabilized and further intracellularly stained with anti-IFN-γBV711,anti-Perforin-AP647,anti-Granzyme B-BV421（Figure3A). Cell viability was determined using Live/Dead fixable viability stain 510 (BD Biosciences, San Jose, CA, USA). Cells were washed and fixed with 2% paraformaldehyde. Cytometer setup and tracking calibration particles were used to ensure that fluorescence intensity measurements were consistent among all experiments. At least 200,000 PBMCs were acquired on a BD FACSCanto II flow cytometer. Gating on forward scatter and side scatter parameters was used to exclude cell debris from the analysis; the forward height and forward area were used to exclude doublets. Data analysis was performed using FlowJo 7.6.1 software version 10.4 (TreeStar, Ashland, OR, USA).
